# Insights into the Structural Patterns in Human Glioblastoma Cell Line SF268 Activity and ADMET Prediction of Curcumin Derivatives

**DOI:** 10.3390/pharmaceutics17080968

**Published:** 2025-07-25

**Authors:** Lorena Coronado, Johant Lakey-Beitia, Marisin Pecchio, Michelle G. Ng, Ricardo Correa, Gerardo Samudio-Ríos, Jessica Cruz-Mora, Arelys L. Fuentes, K. S. Jagannatha Rao, Carmenza Spadafora

**Affiliations:** 1 Center for Molecular and Cellular Biology of Diseases, Instituto de Investigaciones Científicas y Servicios de Alta Tecnología (INDICASAT AIP), Clayton, City of Knowledge, Panama City 0843-01103, Panama; mng@indicasat.org.pa (M.G.N.); rcorrea@indicasat.org.pa (R.C.); gerardo.samudio@utp.ac.pa (G.S.-R.); cspadafora@indicasat.org.pa (C.S.); 2 Sistema Nacional de Investigación (SNI), SENACYT, Panama City 0816-02852, Panama; 3 Center for Biodiversity and Drug Discovery, Instituto de Investigaciones Científicas y Servicios de Alta Tecnología (INDICASAT AIP), Clayton, City of Knowledge, Panama City 0843-01103, Panama; jcruz@indicasat.org.pa (J.C.-M.); afuentes@indicasat.org.pa (A.L.F.); 4 Center for Academic Affairs and Collaboration, Instituto de Investigaciones Científicas y Servicios de Alta Tecnología (INDICASAT AIP), Clayton, City of Knowledge, Panama City 0843-01103, Panama; mpecchio@indicasat.org.pa; 5 PhD Program in Biosciences and Biotechnology, Faculty of Science and Technology, Universidad Tecnológica de Panamá, Panama City 0819-07289, Panama; 6 Department of Biotechnology, Koneru Lakshmaiah Education Foundation (KLEF) Deemed to be University, Vaddeswaram 522302, India; prochancellor@kluniversity.in

**Keywords:** curcumin, curcumin derivatives, central nervous system, glioblastoma, structure–activity relationship, ADMET prediction, mechanism of action, ROS, tubulin, BAX protein

## Abstract

**Background/Objectives**: Curcumin is a promising therapy for glioblastoma but is limited by poor water solubility, rapid metabolism, and low blood–brain barrier penetration. This study aimed to evaluate curcumin and six curcumin derivatives with improved activity against a glioblastoma cell line and favorable absorption, distribution, metabolism, excretion, and toxicity (ADMET) properties. **Methods**: Twenty-one curcumin derivatives were assessed and subjected to in vitro MTT cytotoxicity assays in SF268 glioblastoma and Vero cells. On the basis of the cytotoxicity results, six derivatives with the most favorable characteristics were selected for additional mechanistic studies, which included microtubule depolymerization, mitochondrial membrane potential (ΔΨm), and BAX activation assays. ADMET properties were determined in silico. **Results:** Compounds **2**–**4**, **6**, and **11** demonstrated better activity (IC_50_: 0.59–3.97 µg/mL and SI: 3–20) than curcumin (IC_50_: 6.3 µg/mL; SI: 2.5). Lead derivatives destabilized microtubules, induced ΔΨm collapse, and activated BAX. In silico ADMET prediction analysis revealed that compounds **4** and **6** were the most promising for oral administration from a biopharmaceutical and pharmacokinetic point of view. **Conclusions**: Strategic modifications were made to one or both hydroxyl groups of the aromatic rings of curcumin to increase its physicochemical stability and activity against glioblastoma cell line SF268. Compound **4**, bearing fully protected aromatic domains, was identified as a prime candidate for in vivo validation and formulation development.

## 1. Introduction

Cancer is a group of diseases characterized by the uncontrolled growth and spread of abnormal cells that can invade adjacent tissues and organs [1]. It is one of the leading causes of death worldwide, representing a significant burden for both patients and health care systems [2,3].

According to the International Agency for Research on Cancer (IARC), in 2022, there were approximately 20 million new cancer cases and 9.7 million cancer-related deaths globally [4]. The most common types of cancer include breast, lung, colorectal, and prostate cancer [5]. Furthermore, the incidence of cancer is expected to rise in the coming years due to population aging and other risk factors, such as smoking, unhealthy diets, and physical inactivity [6,7,8]. Within this global cancer landscape, certain less common types, such as central nervous system (CNS) tumors, pose a particular challenge due to their complexity and aggressiveness [9].

CNS tumors are a diverse group of neoplasms that include both primary and metastatic tumors [10,11]. Among primary CNS tumors, glioblastoma is the most aggressive and common type, accounting for approximately 15% of all brain tumors and 60–70% of gliomas. This type of cancer is characterized by rapid growth, invasiveness, and resistance to conventional treatments [12,13,14]. The survival rates for glioblastoma patients remain very low, with a 5-year survival rate of approximately 6.9% [15]. Despite advancements in surgical techniques and cancer therapies, the treatment of glioblastoma has not significantly changed, with the standard approach still being total tumor resection followed by radiotherapy and chemotherapy with temozolomide [12,15].

Glioblastoma presents significant molecular heterogeneity and a highly immunosuppressive environment, further complicating treatment and reducing the effectiveness of emerging therapies, including immune checkpoint inhibitors [15,16,17,18]. Faced with this challenge, the search for new therapeutic alternatives has led to the exploration of natural compounds with anticancer properties. In this context, turmeric (*Curcuma longa*), a plant from the Zingiberaceae family, is widely used in traditional medicine [19,20] for its neuroprotective [21,22], anti-inflammatory [23,24], antitumor, and chemopreventive effects on various types of cancer [25,26]. The bioactive compounds in turmeric, especially curcuminoids such as curcumin, have shown potential effects in the prevention and treatment of various types of cancer, including glioblastoma [27]. Recent studies have suggested that these compounds may inhibit cell proliferation, induce apoptosis, and increase sensitivity to conventional treatments, making them promising candidates for the development of new anticancer therapies [25,28].

On the basis of the potential of curcumin, several preclinical and clinical studies have been conducted in rodents and humans, respectively. These studies reported limitations in the use of curcumin mainly related to its low bioavailability, rapid metabolism, low blood–brain barrier (BBB) permeability, and instability in aqueous solutions, resulting in degradation at physiological pH [29,30].

**Figure 1:**
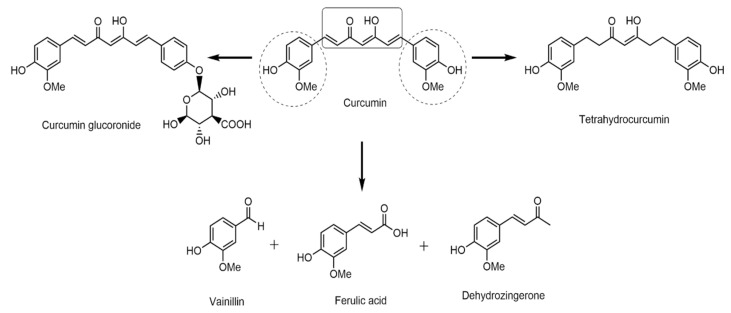
Curcumin structure and regions that are susceptible to transformation. Dash line representing the curcumin site that can suffer transformation meanwhile the arrow indicates the curcumin transformation.

Curcumin (1,7-bis(4-hydroxy-3-methoxyphenyl)hepta-1,6-diene-3,5-dione) is composed of two aromatic ring systems: *o*-methoxy phenolic groups, linked by a chain of seven α- and β-unsaturated carbon atoms, with a β-diketone moiety. This structural characteristic gives curcumin functionalities such as Michael acceptors (olefins or acetylenes conjugated with electron-withdrawing groups), chelating agents, and H-donors [31]. Its molecular formula is C_21_H_20_O_6_, the detailed structure of which is shown in Figure 1. The highlighted curcumin moieties are associated with their polypharmacology and are susceptible to some modifications. Studies in vivo have reported that, depending on the method of administration, curcumin can form different metabolites. The most important metabolite is curcumin glucuronide when administered orally and tetrahydrocurcumin when administered systemically; these metabolites also degrade under biological conditions, producing vanillin, ferulic acid, and dehydrozingerone [32,33]. These transformations affect the biological activities of the compounds [34]. To take advantage of the natural properties of curcumin and improve its chemical characteristics and bioavailability, different substituent groups were incorporated into the phenolic ring region of the molecule through organic reactions, which allowed twenty-one derivatives to be obtained from curcumin.

Curcumin (**1**) and its derivatives (**2**–**22**) were evaluated for their cytotoxic activity against glioblastoma using the SF268 cell line, which represents a stable cell line derived from central nervous system tumors. This makes it a valuable tool for studying how bioactive compounds, such as curcuminoids, can inhibit cell growth, induce apoptosis, or alter key signaling pathways associated with tumor proliferation [35,36,37]. Additionally, we performed mechanistic studies to identify possible molecular targets and pathways associated with the lead compounds. These experiments provided insights into the modes of action of the compounds.

In drug discovery, early ADMET (absorption, distribution, metabolism, excretion, and toxicity) profiling is essential to reduce the high attrition rates in preclinical and clinical development. The above factors are very important for obtaining drug candidates because of their impact on the success or failure of drug development in the clinical phase [38]. However, experimental ADMET trials with many compounds are complex and expensive. To counteract this dilemma, different in silico strategies currently exist to predict ADMET properties before experimental evaluation, facilitating the processing of many compounds and consequently saving time and resources [39]. For this reason, the pharmacokinetics and ADMET of curcumin and six active curcumin derivatives were evaluated in silico before their consideration for in vitro and in vivo studies.

Our aims were as follows: (1) purify selected curcumin derivatives; (2) evaluate the in vitro cytotoxicities and selectivity indices of the active compounds against SF268 glioblastoma and Vero cells to identify the lead compounds; (3) elucidate the mechanism of anticancer action by assessing mitochondrial membrane potential collapse and active Bcl-2-associated X protein (BAX) translocation in treated SF268 cells; (4) predict the physicochemical and ADMET properties of curcumin and its derivatives in silico; and (5) correlate the structure–activity relationships (SARs) of the selected compounds.

## 2. Materials and Methods

### 2.1. Purification of Selected Curcumin Derivatives

Chemical reagents were used as commercially available (Tedia, Fairfield, OH, USA; Sigma Aldrich, St. Louis, MO, USA). Curcumin was obtained from Alfa Aesar (Haverhill, MA, USA), with 95% total curcuminoid content from the turmeric rhizome.

Selected compounds (**4**–**5**, **11**–**14**) were repurified in an Agilent 1260 Infinity II HPLC system equipped with a quaternary pump, an Agilent 1260 Series diode array detector and a normal phase silica gel column (Phenomenex^®^ (Torrance, CA, USA) Luna Silica (2), 250 mm × 10 mm, 5 μm) with a gradient system of n-hexane to ethyl acetate in 20 min at 2 mL/min (Agilent Technologies, Santa Clara, CA, USA) [40].

^1^H and ^13^C NMR spectra were recorded at 500 (^1^H) and 125 MHz (^13^C) on a Jeol JNM-ECZ500R/S1 500 MHz spectrometer in CDCl_3_ (JEOL Ltd. 3-1-2 Musashino, Akishima, Japan) and ^1^H and ^13^C NMR spectra were recorded at 400 (^1^H) and 100 MHz (^13^C) on an Eclipse 400 MHz spectrometer (JEOL, Peabody, MA, USA). Chemical shifts (δ) are reported in parts per million (ppm) from the residual solvent peak and coupling constant (J) in Hz. Proton multiplicity is reported as follows: singlet (s), doublet (d), triplet (t), quartet (quart.), quintet (quint.), septet (sept.), multiplet (m), and broad (br).

#### 2.1.1. (1E,3Z,6E)-3-hydroxy-5-oxohepta-1,3,6-triene-1,7-diyl)bis(2-methoxy-4,1-phenylene) diacetate (**4**)

The product was repurified by HPLC to obtain diester **4**. ^1^H NMR (500 MHz, CDCl_3_) δ 15.86 (s, 1H), 7.62 (d, *J* = 15.8 Hz, 2H), 7.17–7.12 (m, 4H), 7.07 (d, *J* = 8.1 Hz, 2H), 6.57 (d, *J* = 15.8 Hz, 2H), 5.86 (s, 1H), 3.88 (s, 6H), 2.33 (s, 6H). ^13^C NMR (125 MHz, CDCl_3_) δ 183.22, 169.04, 151.48, 141.36, 140.11, 134.08, 124.34, 123.42, 121.23, 111.49, 102.02, 56.04, and 20.84 ppm.

#### 2.1.2. Cyclopentyl (4-((1E,3Z,6E)-3-hydroxy-7-(4-hydroxy-3-methoxyphenyl)-5-oxohepta-1,3,6-trien-1-yl)-2-methoxyphenyl) succinate (**5**)

The product was repurified by HPLC to obtain diester **5**. ^1^H NMR (400 MHz, CDCl_3_): δ 7.60 (dd, *J* = 16, 4 Hz, 2H), 7.19–7.03 (m, 5H), 6.93 (d, *J* = 8 Hz, 1H), 6.52 (dd, *J* = 22, 16 Hz, 1H), 5.83 (s, 1H), 5.19 (m, 1H), 3.95 (s, 3H), 3.87 (s, 3H), 2.92 (t, *J* = 14 Hz, 2H), 2.71 (t, *J* = 14 Hz, 2H), 1.85–1.72 (m, 4H), 1.68–1.58 (m, 4H) ppm.

^13^C NMR (100 MHz, CDCl_3_) δ 184.64, 182.00, 171.91, 170.51, 151.52, 148.17, 146.98, 141.28, 139.57, 134.27, 127.75, 124.44, 123.41, 123.19, 121.96, 121.10, 115.02, 111.65, 109.85, 101.64, 77.80, 56.14, 56.10, 32.79, 32.76, 29.68, 29.57, 29.22, and 23.88 ppm.

#### 2.1.3. Allyl (4-(1E,3Z,6E)-3-hydroxy-7-(4-hydroxy-3-methoxyphenyl)-5-oxohepta-1,3,6-trien-1-yl)-2-methoxyphenyl) succinate (**11**)

The product was repurified by HPLC to obtain diester **11**. ^1^H NMR (500 MHz, CDCl_3_): δ 7.60 (dd, *J* = 16, 6 Hz, 2H), 7.13–7.04 (m, 5H), 6.93 (d, *J* = 8 Hz, 1H), 6.52 (dd, *J* = 22, 16 Hz, 1H), 5.99–5.87 (m, 2H), 5.83 (s, 1H), 5.33 (dd, *J* = 17, 2 Hz, 1H), 5.25 (dd, *J* = 10, 1 Hz, 1H), 4.63 (d, *J* = 6 Hz, 2H), 3.94 (s, 3H), 3.86 (s, 3H), 2.95 (t, *J* = 14 Hz, 2H), 2.79 (t, *J* = 14 Hz, 2H) ppm. ^13^C NMR (100 MHz, CDCl_3_) δ 184.65, 181.96, 171.81, 170.41, 151.49, 148.16, 146.97, 141.26, 139.52, 134.28, 132.11, 127.72, 124.44, 123.39, 123.18, 121.94, 121.07, 118.54, 115.02, 111.63, 109.84, 101.65, 65.65, 56.10, 29.29, and 29.09 ppm.

#### 2.1.4. Diallyl O,O′-(((1E,3Z,6E)-3-hydroxy-5-oxohepta-1,3,6-triene-1,7-diyl)bis(2-methoxy-4,1-phenylene)) disuccinate (**12**)

The product was repurified by HPLC to obtain diester **12**. ^1^H NMR (400 MHz, CDCl_3_): δ 7.61 (d, *J* = 16 Hz, 1H), 7.18–7.01 (m, 8H), 6.56 (d, *J* = 16 Hz, 1H), 5.99–5.86 (m, 2H), 5.85 (s, 1H), 5.33 (dd, *J* = 17, 2 Hz, 2H), 5.24 (dd, *J* = 10, 1 Hz, 2H), 4.63 (d, *J* = 5.8 Hz, 4H), 3.86 (s, 6H), 2.94 (t, *J* = 14 Hz, 4H), 2.79 (t, *J* = 14 Hz, 4H) ppm.

^13^C NMR (100 MHz, CDCl_3_) δ 183.24, 171.78, 170.37, 151.53, 141.40, 140.09, 134.16, 132.12, 124.45, 123.43, 121.19, 118.53, 111.67, 101.91, 65.63, 56.10, 29.29, and 29.09 ppm.

#### 2.1.5. Benzyl (4-((1E,3Z,6E)-3-hydroxy-7-(4-hydroxy-3-methoxyphenyl)-5-oxohepta-1,3,6-trien-1-yl)-2-methoxyphenyl) succinate (**13**)

The product was repurified by HPLC to obtain diester **13**. ^1^H NMR (400 MHz, CDCl_3_): δ 7.60 (dd, *J* = 16, 6.0 Hz, 2 H), 7.36 (s, 5H), 7.19–6.98 (m, 5H), 6.93 (d, *J* = 8 Hz, 1H), 6.52 (dd, *J* = 21, 16 Hz, 2H), 5.93 (s, 1H), 5.83 (s, 1H), 5.17 (s, 2H), 3.94 (s, 3H), 3.84 (s, 3H), 2.96 (t, *J* = 14 Hz, 2H), 2.82 (t, *J* = 14 Hz, 2H) ppm.

^13^C NMR (100 MHz, CDCl_3_) δ 184.65, 181.95, 171.97, 170.39, 151.47, 148.15, 146.96, 141.28, 141.22, 139.53, 135.85, 134.26, 128.73, 128.46, 128.37, 127.70, 124.41, 123.38, 123.18, 121.92, 121.07, 115.01, 111.58, 109.82, 101.66, 66.83, 56.11, 56.07, 29.39, and 29.11 ppm.

#### 2.1.6. Dibenzyl O,O′-(((1E,3Z,6E)-3-hydroxy-5-oxohepta-1,3,6-triene-1,7-diyl)bis(2-methoxy-4,1-phenylene)) disuccinate (**14**)

The product was repurified by HPLC to obtain diester **14**. ^1^H NMR (400 MHz, CDCl_3_) δ 7.62 (d, *J* = 16 Hz, 1H), 7.36 (s, 10H), 7.18–6.95 (m, 8H), 6.56 (d, *J* = 16 Hz, 1H), 5.86 (s, 1H), 5.17 (s, 4H), 3.85 (s, 6H), 2.96 (t, *J* = 14 Hz, 4H), 2.82 (t, *J* = 14 Hz, 4H) ppm.

^13^C NMR (100 MHz, CDCl_3_) δ 183.25, 171.95, 170.35, 151.53, 141.39, 140.11, 135.88, 128.74, 128.46, 128.38, 124.45, 123.44, 121.21, 111.65, 101.91, 66.82, 56.10, 29.41, and 29.14 ppm.

### 2.2. Cell Culture

Grade IV SF268 glioblastoma cells and Vero epithelial cells were obtained from ATCC and cultured in complete medium consisting of Roswell Park Memorial Institute medium (RPMI-1640) supplemented with 0.05% gentamicin (50 mg/mL) and 10% FBS (fetal bovine serum; Gibco, Invitrogen, Carlsbad, CA, USA). Cultures were maintained at 37 °C in a 5% CO_2_ atmosphere and split by trypsin treatment once per week to maintain confluence.

### 2.3. Mammalian Cytotoxicity

For the cytotoxicity assays, Vero cells were cultivated in 96-well plates at 37 °C under a 5% CO_2_ atmosphere using RPMI-1640 medium with 0.05% gentamicin (50 mg/mL) and 10% FBS (fetal bovine serum). Vero cells were allowed to adhere for 24 h before being incubated for 48 h with the samples. During the treatment, dimethyl sulfoxide (DMSO) was used as a negative control, curcumin (**1**) was used as a control, and samples were assayed at four different concentrations. After incubation, MTT (3-(4,5-di-methyl-thiazol-2-yl)-2,5-diphenyl-tetrazolium bromide) was added to each well, and the absorbance at 570 nm was determined 4 h later using a color plate reader. Cytotoxicity was evaluated colorimetrically by calculating the ability of the remaining living Vero cells to reduce the pale yellow MTT to the black–purple formazan product, as previously described [41]. All bioassays were performed in duplicate, and the inhibitory concentration (IC_50_) values were determined using the Data Analysis complement Wizard of Excel 2000 (Microsoft, Seattle, WA, USA).

### 2.4. SF268 Cell Line Citotoxicity

SF268 cancer cells were cultivated in 96-well plates at 37 °C under a 5% CO_2_ atmosphere with sterilized RPMI-1640 supplemented with 0.05% gentamicin (50 mg/mL) and 10% FBS (fetal bovine serum) and allowed to adhere for 24 h [42]. After incubation, the cancer cells were treated with curcumin derivatives dissolved in DMSO at six different concentrations for 48 h and analyzed in duplicate. RPMI without cells was used as a color control, and doxorubicin was used as an inhibition control. After treatment, MTT was added to each well, and the absorbance at 570 nm was determined 4 h later using a color plate reader. All bioassays were performed in duplicate, and the IC_50_ values were determined by the Data Analysis complement Wizard of Excel 2000 (Microsoft, Seattle, WA, USA).

### 2.5. Mechanism of Action

#### 2.5.1. In Vitro Tubulin Polymerization

A Tubulin Polymerization Assay Kit (Porcine tubulin and Fluorescence-based; Cat. # BK011P) (Cytoskeleton Inc., Denver, CO, USA) was used. Neural tubulin (>99% pure cytoskeleton) and tubulin polymerization buffer were mixed with 10 µg/mL curcumin derivatives or the vehicle DMSO at 37 °C in a preheated 96-well plate. The measurement of absorbance at 340 nm was immediately initiated using a Bio-Tek Synergy HT plate reader (one reading per minute for 1 h, according to the kit instructions). The rate of tubulin polymerization (milli-OD340 units per min) was then calculated for each sample from the linear portion of the kinetics curve, and group mean rates were analyzed for statistical significance.

#### 2.5.2. Immunofluorescence Staining of Microtubules in SF268 Cells

SF268 cells were seeded onto 96-well plates and allowed to adhere overnight in complete growth medium (RPMI-1640). The treatments were as follows:

- A (vehicle control): 0.1% DMSO
- B (apoptosis control): 10 µM staurosporine
- C–F (test compounds): curcumin (10 µg/mL), compound **2** (10 µg/mL), compound **6** (10 µg/mL), and compound **11** (10 µg/mL).

After treatment, the cells were rinsed two times with PBS, fixed in 4% paraformaldehyde (15 min), and permeabilized with 0.5% Triton X-100 (Sigma Aldrich, St. Louis, MO, USA) (5 min). Nonspecific binding was blocked with 5% bovine serum albumin (BSA) in phosphate-buffered saline (PBS) for 1 h. Samples were incubated with mouse anti-β-tubulin primary antibody (1:100 dilution in 5% BSA) overnight at 4 °C, washed 3 times with PBS, incubated with Alexa Fluor 488-conjugated secondary anti-mouse antibody (1: 500 dilution) for 2 h at room temperature in the dark, and washed 3 times with PBS. Nuclei were counterstained with Hoechst 33342 (15 min), washed 2 times with PBS, and left in PBS at 4 °C in the dark.

Fluorescence images were acquired with an Olympus IX70 microscope (Olympus, Tokyo, Japan) using a 60× objective with identical exposure settings for all conditions. Microtubule density and organization were acquired and analyzed using β-tubulin signals in HCImage Hamamatsu (version 4.8) and ImageJ (version 1.54).

#### 2.5.3. Immunofluorescence Staining of BAX in SF268 Cells

SF268 cells were seeded onto 96-well plates and allowed to adhere overnight in complete growth medium (RPMI-1640). The treatments were as follows:

- A (vehicle control): 0.1% DMSO
- B (apoptosis control): 10 µM staurosporine
- C–F (test compounds): curcumin (10 µg/mL), compound **2** (10 µg/mL), compound **6** (10 µg/mL), and compound **11** (10 µg/mL).

After treatment, the cells were rinsed two times with PBS, fixed in 4% paraformaldehyde (15 min), and permeabilized with 0.5% Triton X-100 (5 min). Nonspecific binding was blocked with 5% BSA in PBS for 1 h. Samples were incubated with mouse anti-active Bax (clone 6A7; 1:100 dilution in 5% BSA) overnight at 4 °C, washed 3 times with PBS, incubated with Alexa Fluor 555-conjugated secondary anti-rabbit antibody (dilution 1:500) for 2 h at room temperature in the dark, and washed 3 times with PBS. Nuclei were counterstained with Hoechst 33342 (Abcam Inc., Waltham, MA, USA) (15 min), washed 2 times with PBS, and left in PBS at 4 °C in the dark.

Fluorescence images were acquired with an Olympus IX70 microscope using a 60× objective with identical exposure settings for all conditions. The intensity of active Bax per cell was acquired and analyzed using ImageJ (version 1.54).

#### 2.5.4. Mitochondrial Membrane Potential (Δψ) Measurements

Changes in membrane potential were measured by staining cultured cells with the mitochondrial selective dye 3,3′-dihexyloxacarbocyanine (DiOC_6_) at 10 nM (Thermo Fisher, Waltham, MA, USA). The samples were incubated at 10 μg/mL for 6 h at 37 °C and then stained for 20 min with DiOC_6_ in the dark. Following incubation, the cells were washed, and the green fluorescence intensity of the DiOC_6_-stained cells was analyzed with a fluorometer immediately after staining. Incubation for 1 h with the mitochondrial membrane disruptor CCCP, carbonyl cyanide *m*-chlorophenylhydrazone (50 μM final concentration), was used as a positive control.

#### 2.5.5. Intracellular Reactive Oxygen Species Production

Intracellular ROS formation was measured by fluorometry with the reactive dye chloromethyl dichlorodihydrofluorescein diacetate (DCFDA; Molecular Probes). Hydrogen peroxide, a potent oxygen radical inducer, was used as a positive control at a final concentration of 100 μM. Cultures were incubated with curcumin derivatives at 10 μg/mL for 3 h, after which the ROS-specific fluorescent dye DCFDA solution was added to the cells. After 30 min, fluorescence was measured at a wavelength of 485 nm (excitation)/535 nm (emission) using a microplate reader (Molecular Devices, San Jose, CA, USA).

### 2.6. Prediction of Physicochemical and ADMET Properties and Pharmacokinetic Parameters

#### 2.6.1. Prediction of Physicochemical Properties

The SwissADME and ADMET Predictor^®^ versions 10.4 and 11.0 (Simulations Plus Inc., Lancaster, CA, USA) programs were used to predict the physicochemical properties of curcumin and its selected derivatives. Lipinski’s and Veber’s rules were used to analyze which compounds met the established parameter ranges. Both rules are used to predict oral drug delivery [43,44]. For Lipinski’s rule, a molecular weight (MW) of less than 500 g/mol, no more than 5 hydrogen donor bonds (HDBs), 10 hydrogen acceptor bonds (HABs), and an octanol/water partition coefficient (logP) of less than 5 are assessed. Compounds that meet these four requirements are likely to be effectively absorbed from the intestines into the blood, successfully exerting passive diffusion across cell membranes. For Veber’s rule, a compound with 10 or fewer rotational bonds (RBs) and a polar surface area (TPSA) less than or equal to 140 Å^2^ is likely to have good oral bioavailability.

#### 2.6.2. Prediction of ADMET Properties and Pharmacokinetic Parameters

ADMET Predictor^®^ software versions 10.4 and 11.0 (Simulations Plus Inc., Lancaster, CA, USA) were used as an in silico prediction and simulation tool. Biopharmaceutical and pharmacokinetic analyses were used to evaluate the absorption, distribution, metabolism or biotransformation, excretion, and toxicity of curcumin and its derivatives. Simulations of curcumin (**1**) and its derivatives (**2**–**22**) were performed with a single human oral dose of 10 mg over 168 h. Some of the ADMET properties that were evaluated for each compound include blood–brain barrier (BBB) permeability, cytochrome P450 isoenzymes (CYP1A2, CYP2C9, CYP2C19, CYP2D6, and CYP3A4) activity, and P-glycoprotein (P-gp) transportation, among others. For each compound, various pharmacokinetic parameters, namely, the absorbed fraction (Fa%), bioavailable fraction (Fb%), volume of distribution in humans (Vd), and half-life (t_1/2_), among others, were assessed.

### 2.7. Statistical Analysis

Statistical analysis of the cytotoxicity assays results was performed independently. The data are presented as the IC_50_ ± standard error (SE). IC_50_ values were calculated using the Data Analysis add-in Wizard of Excel 2000 by fitting the dose–response curve to a sigmoidal model (Microsoft, Seattle, WA, USA). For comparisons among multiple treatment groups, one-way analysis of variance (ANOVA) was conducted using GraphPad Prism 9.0 (GraphPad Software, San Diego, CA, USA). Bar graphs depict the mean values ± SEs.

## 3. Results

### 3.1. Curcumin Derivatives

**Figure 2:**
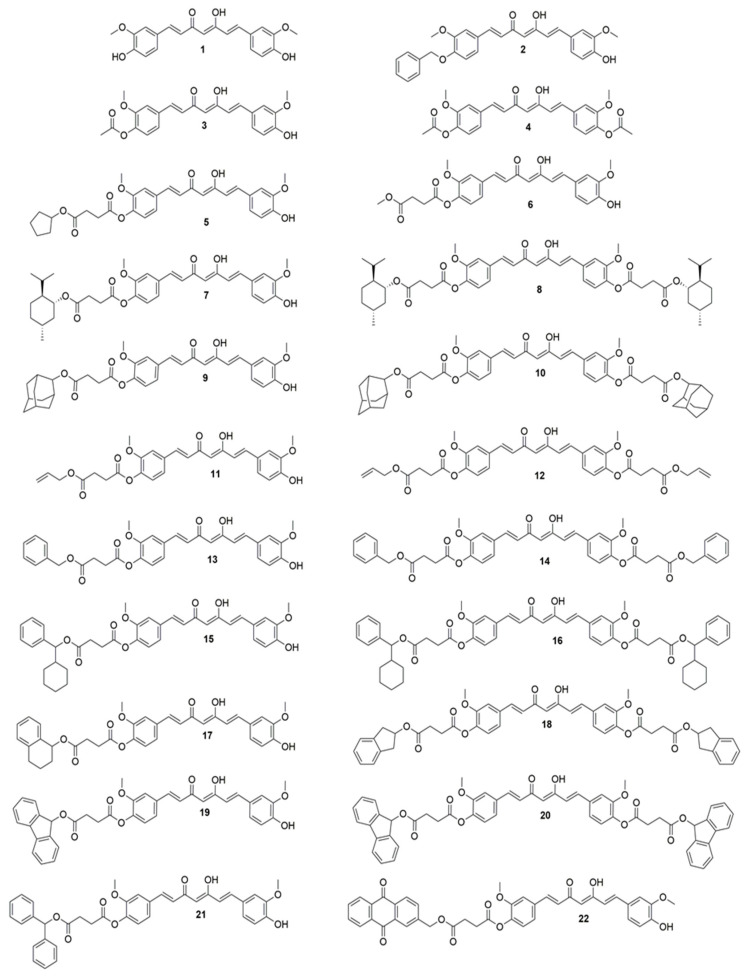
Curcumin and its derivatives.

The chemical structures of curcumin and the curcumin derivatives **2**–**22** are shown in Figure 2. These derivatives were previously synthesized by our group [30,40]. Compounds **2**–**4** were synthesized by etherification and esterification of curcumin. Compounds **5**–**22** were synthesized by forming a succinate (linker) and then esterifying curcumin. Some compounds (**4**–**5**, **11**–**14**) were repurified before their biological activities were evaluated (Supplementary Data). All the compounds (**1**–**22**) were prepared to evaluate their biological activities.

### 3.2. SF268 Cell Line Cytotoxic Activities of the Curcumin Derivatives In Vitro

The cytotoxic activities of curcumin (**1**) and its derivatives (**2**–**22**) in the glioblastoma cell line SF268 were evaluated via a screening assay using an MTT (3-(4,5-dimethylthiazol-2-yl)-2,5-diphenyltetrazolium bromide) protocol. The effects of a single concentration (10 µg/mL) of each compound on the SF268 cancer cell line were assessed for 48 h.

**Figure 3:**
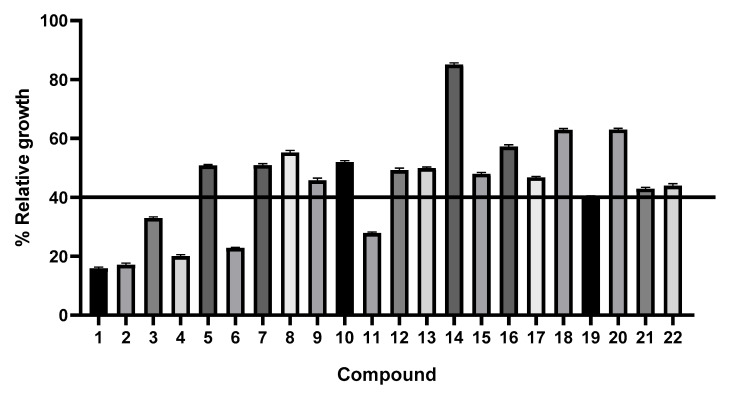
Evaluation of the effects of curcumin (**1**) and its derivatives (**2**–**22**) on the SF268 CNS cancer cell line by the MTT assay. Each experiment was repeated three times, and data are plotted as bar graphs with error bars.

All the compounds (**1**–**22**) were screened for their cytotoxic activity in the glioblastoma cell line. The results obtained from the bioassay are summarized in Figure 3. Those with a percentage of relative growth (%RG) of 40% or less, with some specific exceptions for SAR structure–activity analysis, were selected to determine IC_50_ values.

Compound **11** exhibited the most potent inhibitory effect on the cancer cells in vitro, with an IC_50_ value of 0.59 µg/mL and a selectivity index (SI) of 20.17, which were better than the values presented by curcumin, with an IC_50_ of 6.3 and an SI of 2.52 (Table 1).

The results showed that compounds **2**–**4**, **6**, **11**, and **19** were remarkably active in inhibiting the growth of the cancer cells, with a low toxicity to normal healthy cells, presenting an SI greater than that of curcumin (Table 1).

### 3.3. Mechanism of Action Studies

Six different compounds (**1**–**4**, **6**, **11**) were selected to study the biological effects that occur in the cells due to the action of the curcumin derivatives: curcumin (**1**) was used as a reference control, and the compounds (**2**–**4**, **6**, **11**), with SIs of 3, 13.24, 17.16, 15.45, and 20.17, respectively. These active compounds cover a range of specificities for the cancer cells, varying from moderate to highly selective.

#### 3.3.1. Effects of Curcumin on SF268 Cell Microtubules

**Figure 4:**
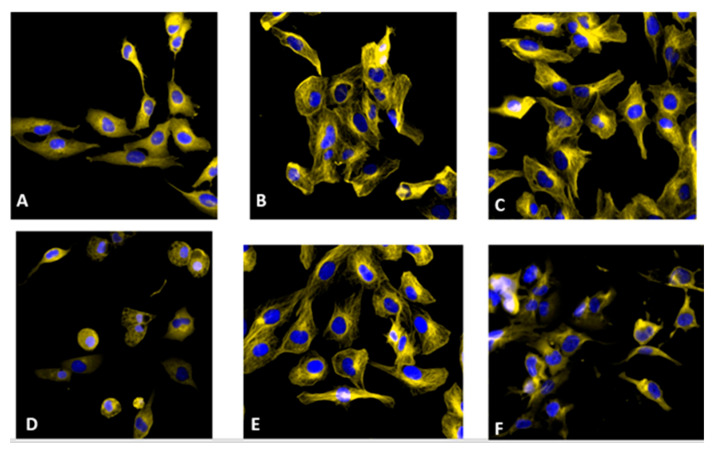
Immunofluorescence staining of microtubules in SF268 cells. Cells were treated for 12 h with 0.5% DMSO as an untreated control (**A**), 10 μM staurosporine (**B**) as an apoptosis control, and the following curcumin derivatives (10 μg/mL): curcumin (**C**), compound **2** (**D**), compound **6** (**E**), and compound **11** (**F**). The cells were then stained with a β-tubulin antibody (yellow) and Hoechst dye (blue) to visualize the nucleus.

Tubulins are key cytoskeletal network components essential for various cellular functions, including mitosis [45]. The effects of curcumin on the interphase microtubules of SF268 cells were examined using immunofluorescence microscopy (Figure 4). SF268 cells were exposed to media containing either DMSO as a control or curcumin derivatives at 10 μg/mL for 24 h, after which the microtubules were visualized with an α tubulin antibody (yellow), and the cell nuclei were stained with Hoechst (blue). The control cells (A) presented typical interphase microtubule organization. A significant reduction in microtubule density occurred after all the treatments but was more evident after treatment with compounds **2**, **6**, and **11** (D,E,F). A significant reduction in the number of microtubules at the periphery of the cells was apparent, and the central networks were disorganized. These results suggest that treatment with the curcumin derivatives disrupts microtubule assembly inside cells, which is essential for the formation of the mitotic spindle and the segregation of condensed chromosomes.

#### 3.3.2. Curcumin Derivatives Affect Tubulin Polymerization

**Figure 5:**
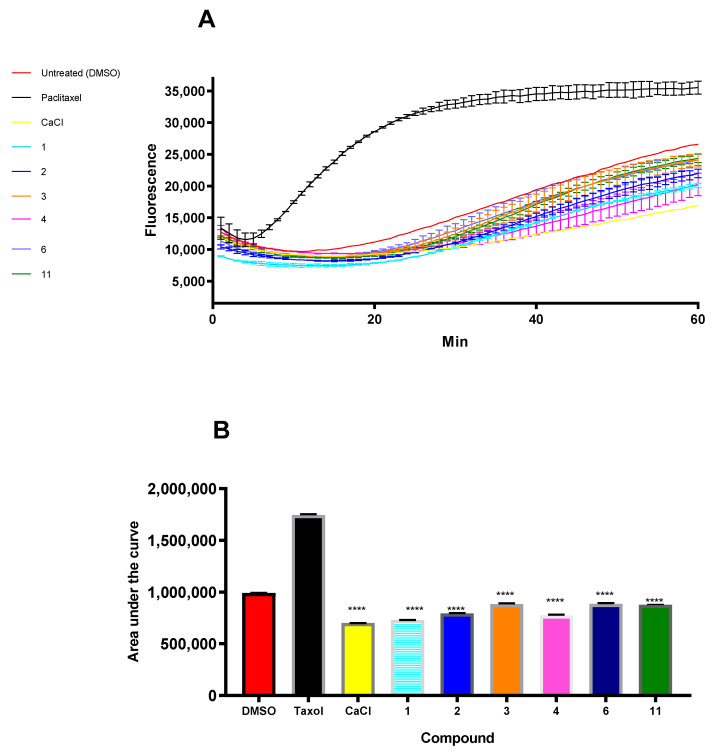
(**A**) Tubulin polymerization of compounds **1**, **2**, **3**, **4**, **6**, and **11**. Tubulin polymerization was monitored by the increase in fluorescence at 360 nm (excitation) and 420 nm (emission) for 1 h at 37 °C. Paclitaxel and calcium chloride were used as positive controls, whereas 0.1% DMSO was used as a negative control. (**B**) AUC for the tested compounds and positive and negative controls. **** *p* ≤ 0.0001.

Because curcumin depolymerized the microtubules in the SF268 cells, we examined the effects of curcumin on microtubule polymerization in vitro. We used a fluorescence cell-free-based assay to analyze the ability of curcumin to inhibit tubulin polymerization into microtubules in vitro. The curcumin derivatives inhibited the rate of tubulin assembly in a way similar to that of calcium chloride, which was used as a positive control for tubulin depolymerization (Figure 5A).

The tubulin polymerization activities were determined by measuring fluorescence and recording the area under the curve (AUC). Increasing fluorescence indicates increasing polymerization activity, whereas decreasing fluorescence indicates greater depolymerization activity. Paclitaxel, which stabilizes polymerized tubulin, caused an increase in the AUC. On the other hand, the destabilizing compound calcium chloride caused a significant decrease in the AUC compared with that of the negative control, inhibiting tubulin polymerization.

Compared with the DMSO-untreated (UT) control, all the compounds tested (**1**–**4**, **6**, and **11**) caused a significant decrease in fluorescence, with a pattern similar to that of the calcium chloride-positive control. These results suggest that all the curcumin derivatives inhibited tubulin polymerization (Figure 5B).

#### 3.3.3. Curcumin Derivative Treatment Affects the Mitochondrial Membrane Potential

**Figure 6:**
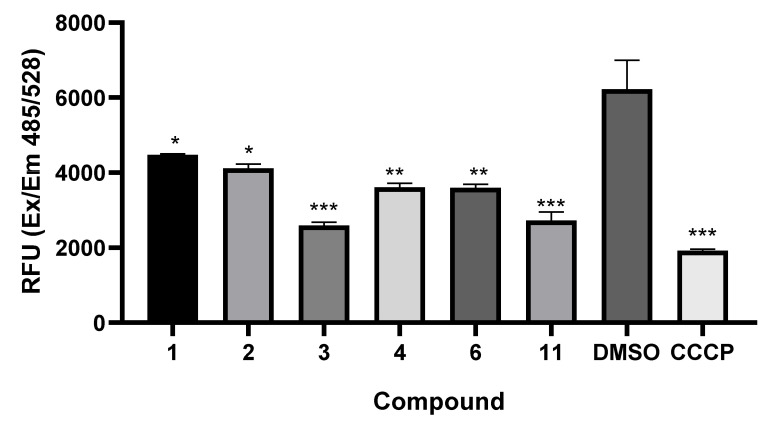
Changes in mitochondrial membrane potential caused by the compounds. Fluorometry was used to measure the mitochondrial effects of compounds that inhibited cell growth in culture. A membrane disruptor (CCCP) was added as a positive control, and untreated cultures (DMSO) were used as a negative control. All compounds were tested at 10 µg/mL for 6 h in triplicate, *n* = 3; * *p* ≤ 0.05, ** *p* < 0.01, *** *p* < 0.005.

Changes in the mitochondrial membrane potential, a common hallmark of programmed cell death, were analyzed in samples with promising activities toward CNS cell line growth. We examined the effects of these compounds on the mitochondrial membrane potential using the dye DiOC_6_, a fluorophore that accumulates rapidly and selectively inside mitochondria depending on the membrane potential. The results of adding 10 µg/mL of each compound are shown in Figure 6. We found that treatment with all the selected compounds tested (**1**–**4**, **6**, and **11**) disrupted the mitochondrial membrane potential, as evidenced by a significant decrease in the proportion of cells with greater fluorescence intensity than that of the DMSO vehicle control. The most disruptive compounds were **3** and **11**, which presented the lowest fluorescence intensity compared with that of the untreated control.

#### 3.3.4. Curcumin Derivatives Do Not Result in Reactive Oxygen Species (ROS) Production

Generally, a decrease in ΔΨm is associated with the production of ROS resulting from mitochondrial destabilization. Therefore, we assayed the intracellular production of reactive oxygen species (ROS) for the selected compounds (**1**–**4**, **6**, and **11**) and compared the level of ROS production between treated and untreated cells.

**Figure 7:**
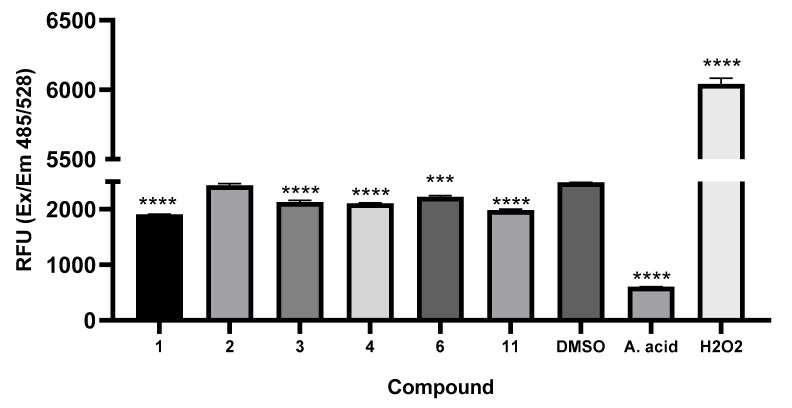
Effects of curcumin derivatives on ROS generation. Cells were treated with curcumin derivatives at 10 μg/mL for 3 h. ROS production was determined using the DCFH-DA assay. Data are presented as the means ± SEMs (*n* = 3). *** *p* ≤ 0.001, **** *p* ≤ 0.0001.

Intracellular ROS production was tested following the treatment of SF268 cells with the curcumin derivatives at 10 μg/mL by fluorometry after staining with DCFDA. The results did not reveal an increase in the generation of ROS when the cells were incubated with the samples for 3 h. In contrast, the cells treated with H_2_O_2_ as a positive control presented distinct ROS production, and an expected decrease was found after treatment with ascorbic acid (a well-known antioxidant) (Figure 7). Hence, our results suggest that treatment with the curcumin derivatives **2**–**4**, **6**, and **11** does not result in ROS production, indicating that cell death is not caused by oxidative stress.

#### 3.3.5. Treatment with Curcumin Derivatives Increases Active Bax Levels in SF268 Cancer Cells

**Figure 8:**
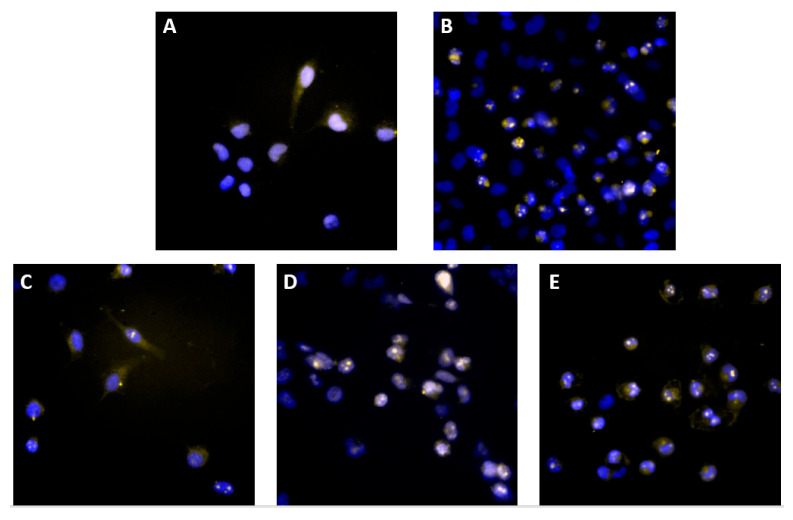
Immunofluorescence staining of active BAX in SF268 cells with a Bax 6A7 antibody. Cells were treated for 12 h with 0.1% DMSO as an untreated control (**A**) or with the following (10 μg/mL): curcumin (**B**), compound **2** (**C**), compound **6** (**D**), and compound **11** (**E**). The cells were then stained with anti-BAX antibody (yellow) and Hoechst dye (blue) to visualize the nucleus.

The Bcl-2 protein family consists of both proapoptotic (Bax, Bad, Bid, and Bim) and antiapoptotic (Bcl-2 and Bcl-XL) proteins that regulate mitochondrial outer membrane integrity, cytochrome c release, caspase activation, and apoptosis. Therefore, we measured the amount of Bax protein by immunofluorescence using an anti-active Bax antibody after the curcumin derivative treatment for 24 h. The fluorescence images revealed that, compared with the DMSO untreated control, treatment with the curcumin derivatives increased the amount of the proapoptotic protein Bax (Figure 8). These data suggest that Bcl-2 family members play a role in the curcumin-induced apoptosis of SF268 cancer cells.

### 3.4. Prediction of Physicochemical and ADMET Properties and Pharmacokinetic Parameters

#### 3.4.1. Prediction of Physicochemical Properties

Six curcumin derivatives (**2**–**4**, **6**, **11**, and **19**) were selected according to their active selectivity indices against SF268 cells. Predictions of the physicochemical properties of curcumin and its derivatives were obtained and are shown in Table 2**.** The solubility of these compounds in water was predicted to be between 1.13 × 10^−5^ and 0.05 mg/mL, the diffusion coefficient was predicted to be between 0.49 and 0.66 cm^2^/s × 10^5^, and the brain/blood partition coefficient (log BB) was predicted to be between −1.26 and −0.82. Only compounds **1**–**4** and **6** have a molecular weight less than 500 g/mol and an octanol/water partition coefficient (logP) between 3.51 and 5.27; they present between 6 and 9 HAB and between 1 and 3 HDB. They have a TPSA ranging from 85.22 to 128.59 Å^2^ and between 7 and 13 RB.

#### 3.4.2. Prediction of ADMET Properties and Pharmacokinetic Parameters

In silico, individual analyses of the absorption, distribution, metabolism, excretion, and toxicity processes were performed after a human oral dose of 10 mg of curcumin and its derivatives. The Fa% and Fb% of compounds **3**–**4** and **6** were greater than 50% but less than those of compound **1**. The compound with the greatest unbound fraction in humans (FU%) relative to plasma proteins and albumin was predicted to be compound **4**, whose result exceeded that reported for compound **1**. Curcumin and six curcumin derivatives (**2**–**4**, **6**, **11**, and **19**) were predicted to be likely to slowly cross the blood–brain barrier and not leak through hERG-encoded potassium channels. Curcumin and six curcumin derivatives (**2**–**4**, **6**, **11**, and **19**) were predicted to be inhibitors and substrates of P-glycoprotein and breast cancer resistance protein (BCRP). As shown in Table 3, compounds **4** and **19** were predicted to be CYP3A4 substrates, and compounds **2**, **6**, **11**, and **19** were predicted to be CYP3A4 inhibitors, while compounds **19** and **3** were predicted to be CYP2D6 and CYP2C9 inhibitors, respectively. All the compounds were predicted to be CYP1A2 inhibitors. Additionally, curcumin and six curcumin derivatives (**2**–**4**, **6**, **11**, and **19**) were found to interact with organic cation transporter 2 (OTC2). The results of the ADMET properties are shown in Table 3 and Table 4. According to the in silico Ames test, compounds **2**, **4**, **6**, **11**, and **19** are not expected to cause mutagenicity in DNA. According to our results from the in silico assays shown in Table 4, these compounds exhibit decreased toxicity to higher organisms (*T. pyriformis* toxicity > MT > D. magna toxicity > ORCT > ORAT LD50) [39].

**Figure 9:**
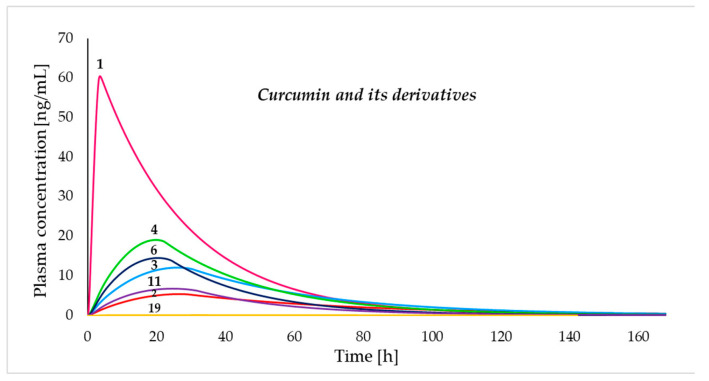
Simulated human plasma concentration versus time after a single human oral administration of 10 mg of each compound using ADMET Predictor^®^ software version 11.0.

Table 5 shows the results of the prediction of the pharmacokinetic properties after a single human dose of each compound (Figure 9), where the compounds presented maximum plasma concentrations between 0.01 and 60.45 ng/mL. In addition, the time to reach maximum blood (Tmax) and AUC values varied between 3.59 and 26.55 h and between 1.00 and 1660.82 ng-h/mL, respectively. The clearance (Cl) of these compounds was between 5.56 and 30.06 L/h. In addition, the Vd and t_1/2_ values recorded for each compound are presented in Table 5.

## 4. Discussion

Curcumin (**1**) and its derivatives (**2**–**22**) represent a promising class of natural-product-inspired agents with potential cytotoxic activity against glioblastoma. Native curcumin’s limitations (poor water solubility, rapid metabolism, and low blood–brain barrier penetration) can be overcome through modifications that optimize its physicochemical and biopharmaceutical properties [46]. Here, we assess how these structural modifications can enhance drug-likeness, in silico ADMET profiles, and apoptotic markers in SF268 cells.

In this study, we worked with twenty-one curcumin derivatives (**2**–**22**) that were classified into two groups: monofunctionalized and difunctionalized. The first group comprised compounds **2**–**7**, **9**, **11**, **13**, **15**, **17**, **19**, and **21**. The second group comprised compounds **8**, **10**, **12**, **14**, **16**, **18**, **20**, and **22**. The substituent groups were acyclic, cyclic, acyclic aromatic, and cyclic aromatic. With these changes in the structure of curcumin, we sought to improve the compound’s stability by protecting its degradation-prone sites while maintaining or even improving its chemical properties against cancer.

We evaluated curcumin and its derivatives (**2**–**22**) for their effects on SF268 cancer cell viability after 48 h. Compounds **2**–**4**, **6**, **11**, **19**, **21**, and **22**, which showed a relative growth (RG) percentage of 40% or less, were selected for subsequent IC_50_ and selectivity index (SI) calculations. While compounds **5**, **8**, and **12**–**14** did not meet this 40% RG threshold, their inclusion in the structure–activity relationship (SAR) analysis provided valuable comparative insights. Our findings suggest that the molecular size of a ligand is a key determinant in reducing %RG. For example, compound **6**, a monofunctionalized derivative with a small ligand, achieved a satisfactory %RG similar to the difunctionalized compound **4**. In contrast, its analogue, compound **5**, which is also monofunctionalized but features a larger ligand, did not meet the threshold. This pattern was reinforced by compounds **7**–**8** and **9**–**10**, where identical linkers and ligands, regardless of mono- or difunctionalization, did not influence %RG. Further supporting the role of molecular size, compound **11** (monofunctionalized with a small ligand) showed superior results compared to compound **12**. Despite sharing the same linker and small ligand, compound **12** is difunctionalized, making it structurally larger, and it did not achieve a satisfactory %RG. A similar trend was observed for compounds **13** and **14**, where neither mono- nor difunctionalization yielded a satisfactory %RG, again suggesting that the overall molecular bulk might be a limiting factor. These results consistently indicate that smaller molecules exhibit a greater capacity to reduce %RG, irrespective of whether they are mono- or difunctionalized.

The cytotoxic effects of the curcumin derivatives were analyzed to identify more active compounds with better structural stabilities than curcumin.

In terms of the SI, compound **11** showed the best activity, with an SI of 20.17, followed by compounds **4**, **6**, **19**, and **3**, with SIs of 17.16, 15.45, 13.71, and 13.24, respectively. The high SIs of these compounds reflect both their potent cytotoxicity against SF268 cells and their relatively low cytotoxicity against Vero cells. The curcumin derivatives **3**, **4**, **6**, **11**, and **19** had better SIs against glioblastoma cancer cells than the curcumin control. In contrast, compound **2** had a moderate SI, comparable to that of curcumin.

The six curcumin derivatives (**2**–**4**, **6**, **11**, and **19**) that exhibited SIs comparable to or greater than that of native curcumin were evaluated by ADMET profiling in silico to identify the candidates suitable for oral administration (Table 6).

From a biological perspective, on the basis of our results, the mechanism of action of the selected curcumin derivatives tested (**2**–**4**, **6**, and **11**) appears to be related to the mitochondrial apoptotic process. In this type of regulated cell death, the defining event is mitochondrial outer membrane permeabilization or collapse.

Changes in the mitochondrial membrane potential are an early hallmark of intrinsic apoptosis pathways. Our results revealed a collapse in the mitochondrial membrane potential of SF268 cancer cells treated for as little as 3 h with the selected compounds **1**–**4**, **6**, and **11**. The degree of membrane depolarization appeared to be partially related to the IC_50_ in SF268 cancer cells, with a lower potential in the cases with an IC_50_ < 1, which is very similar to that observed with the use of the mitochondrial uncoupler CCCP as a positive control. In other words, we observed an activity–effect relationship where the more cytotoxic (lower IC_50_) a compound is, the more effectively it disrupts mitochondrial integrity.

Importantly, although a decrease in ΔΨm is commonly associated with ROS production resulting from mitochondrial destabilization, our results suggest that the curcumin derivative treatments did not result in ROS production, indicating that cell death was not caused by oxidative stress. This could indicate a mechanism different from that of commonly used anticancer treatments, which usually generate high levels of oxidative stress that result in nonspecific toxicity.

Bax, a proapoptotic member of the Bcl-2 family, is a cytosolic protein that inserts into mitochondrial membranes upon the induction of cell death. The complete collapse of ΔΨm is followed by the association of Bax with mitochondria [47]. Our results revealed an increase in Bax-associated fluorescence after the disruption of the mitochondrial membrane potential in cancer cells treated with the curcumin derivatives compared with the DMSO control. Given that many glioblastoma lines overexpress the antiapoptotic proteins Bcl-2 or Bcl-XL [48,49], the ability of our derivatives to overcome this resistance checkpoint via Bax insertion may prove especially valuable in treating refractory tumors.

The assembly dynamics of spindle microtubules are crucial for chromosome segregation during mitosis. Many anticancer drugs inhibit mitosis at metaphase/anaphase by suppressing microtubule dynamics. We found that the selected curcumin derivatives induce the disassembly of microtubules. Interestingly, the tubulin polymerization patterns of the derivatives were similar to those of the inhibitor control. These findings suggest that the curcumin derivatives interact with tubulin in highly proliferating cancer cells, inhibiting microtubule assembly [50].

Importantly, promising compounds should exert their biological activity through different pathways to decrease the probability of resistance development. Our results suggest that the curcumin derivatives **2**–**4**, **6**, and **11** act by different pathways: a mitochondrial apoptotic pathway independent of ROS and the inhibition of cell proliferation with the inhibition of tubulin polymerization. Our results suggest that the curcumin derivatives **2**–**4**, **6**, and **11**, with high levels of selectivity for SF268 cancer cells, may offer interesting alternatives to the treatments currently used for this disease.

While the biological assays revealed apoptotic mechanisms and tubulin inhibition, we next evaluated the physicochemical and ADMET properties of curcumin and the selected derivatives and predicted the corresponding pharmacokinetic profiles in humans. We followed SI-based selection and SAR-driven optimization to ensure that these candidates satisfied key drug-likeness and safety criteria. The physicochemical results indicate that compounds **1**–**4** and **6** adhere to Lipinski’s rule, which means that these compounds could have good oral absorption and permeability through passive diffusion. Compounds **11** and **19** do not adhere to Lipinski’s rule, which suggests that mediated active transport is necessary by the oral route because of their low water solubilities and molecular weights of more than 500 g/mol. Compound **19** also presented a high logP, suggesting a potential limitation in terms of passive diffusion and oral absorption. Additionally, according to Veber’s rule, compounds **1**–**3** are highly likely to have good oral bioavailability and permeability. The ADMET results suggest that compounds **1**, **4**, and **6** have a well-absorbed fraction (Fa%) and a bioavailable fraction (Fb%). Curcumin and six curcumin derivatives (**2**–**4**, **6**, **11**, and **19**) could block the efflux transporters that pump medications out of cells. In general, all the compounds have the advantage of not leaking through hERG-encoded potassium channels, which are expressed in the heart, indicating that they would not cause cardiac toxicity. According to the Ames test, compounds **2**, **4**, **6**, **11**, and **19** did not introduce Ames toxicity, indicating that it is unlikely that they will result in cell mutations. Although compound **3** showed considerably good physicochemical properties, it is predicted to cause toxicity according to the Ames assay, which is not favorable considering that this could affect the safety and efficacy of this compound. Furthermore, none of the compounds resulted in the accumulation of phospholipids, so there would be no such toxicity. The prediction of the pharmacokinetic profiles after the simulation of the oral administration of compounds **1**–**4**, **6**, and **11** revealed a volume of distribution of less than 500 L, indicating that there would be little drug in tissues. Compounds **1**, **4**, and **6** presented lower distribution volumes, with shorter half-lives. According to the biopharmaceutical analysis, compounds **4** and **6** presented better overall properties than did the other compounds in the in vitro and in vivo studies, and compound **11**, owing to its high biological activity and acceptable ADMET properties, should also be considered.

Because curcumin suffers from biotransformation in the aromatic region, resulting in a loss of biological activity, a structure–activity relationship (SAR) was developed for compounds **4**, **6**, and **11**. Protecting these regions could improve the stability and preserve the properties of the molecules. Compounds **6** and **11** have one phenolic ring without protection that can be susceptible to transformation; meanwhile, compound **4** has both aromatic regions protected. Also, compound **4** penetrates the cellular membrane more easily and is more easily metabolically activated than the parent molecule. For this reason, compound **4** was considered the best candidate for further study of its cytotoxic activity against cancer.

## 5. Conclusions

Twenty-one curcumin derivatives were evaluated against SF268 cancer cells, and the results suggest that compounds **2**–**4**, **6**, and **11**, the selected derivatives, induce cell apoptosis. The results also provide evidence for the induction of mitochondrial apoptotic events by the compounds independent of reactive oxygen species, which are associated with the regulation of the Bax protein, and also by affecting the stabilization and polymerization of cell tubulin. These data reveal that curcumin derivatives may be promising therapeutic agents for the treatment of brain cancer by decreasing the number of cancer cells through the induction of apoptosis without generating oxidative stress.

According to the results of the physicochemical and ADMET prediction analyses, compounds **4** and **6** presented better overall properties than the other compounds in vitro and in vivo, and compound **11**, owing to its high biological activity and acceptable ADMET properties, is an important candidate for evaluating other routes of administration as well as new drug delivery systems, such as vectors.

Finally, a structure–activity relationship analysis was performed between monofunctionalized and bifunctionalized derivatives, with and without linkers. It was observed that mono- versus difunctionalization did not affect growth inhibition; instead, molecular size was the critical factor. The smallest compounds (**2**–**4**, **6**, and **11**) exhibited the most potent inhibitory effects. Compound **4**, bearing fully protected aromatic domains, is a prime candidate for in vivo validation and formulation development.

## Figures and Tables

**Table 1:** Growth screening (%RG), IC_50_ values, and selectivity indices of the curcumin derivatives in SF268 and Vero cells.

Sample ID	Growth Screening (%RG)	IC_50_ SF268 (µg/mL)	IC_50_ VERO (µg/mL)	Selectivity Index (SI)
1	15.92	6.3	15.9	2.52
2	17.09	3.966	11.9	3
3	32.94	0.88	13.2	13.24
4	20.02	2.45	42.1	17.16
5	50.81	N/A	12.6	N/A
6	22.79	0.8332	12.9	15.45
7	50.92	N/A	N/A	N/A
8	55.22	N/A	N/A	N/A
9	45.56	N/A	N/A	N/A
10	51.98	N/A	N/A	N/A
11	27.88	0.59	11.9	20.17
12	49.2	N/A	N/A	N/A
13	49.88	N/A	N/A	N/A
14	85.07	N/A	N/A	N/A
15	47.93	N/A	N/A	N/A
16	57.17	N/A	N/A	N/A
17	46.78	N/A	N/A	N/A
18	62.91	N/A	N/A	N/A
19	40.04	1.109	15.2	13.71
20	62.95	N/A	N/A	N/A
21	42.95	36.4	46	1.26
22	43.95	58.87	72.8	1.19

N/A = Nonapplicable.

**Table 2:** Predictions of the physicochemical properties of curcumin and its derivatives analyzed using ADMET Predictor^®^ and SwissADME.

Physicochemical Property		Curcumin and Derivatives						
		1 *	2	3	4	6	11	19
Formula **		C_21_H_20_O_6_	C_28_H_26_O_6_	C_23_H_22_O_7_	C_25_H_24_O_8_	C_26_H_26_O_9_	C_28_H_28_O_9_	C_38_H_32_O_9_
**Water solubility (mg/mL)**		0.04	4 × 10^−3^	3 × 10^−3^	5 × 10^−3^	5 × 10^−3^	3 × 10^−3^	1.13 × 10^−5^
**Diffusion coefficient** **(cm^2^/s × 10^5^)**		0.66	0.57	0.62	0.59	0.57	0.55	0.49
**Log BB**		−0.82	−0.83	−0.89	−1.12	−1.10	−1.23	−1.26
**Lipinski’s Rule**	MW (g/mol) **	368.38	458.51	410.42	452.46	482.49	508.52	632.67
	LogP	3.51	5.27	3.93	3.79	3.70	4.02	7.06
	HAB **	6	6	7	8	9	9	9
	HDB **	3	2	2	1	2	2	2
	Complies with Lipinski’s Rule	Yes	Yes	Yes	Yes	Yes	No	No
**Veber’s Rule**	TPSA (Å^2^) **	96.22	85.22	102.29	108.36	128.59	128.59	128.59
	RB ***	7	10	9	11	13	15	14
	Complies with Veber’s Rule	Yes	Yes	Yes	No	No	No	No

(*) Curcumin (**1**) was used as a control in this study. (**) Results were compared in both ADMET Predictor^®^ and SwissADME, and the results are the same. (***) Results were obtained using only SwissADME software. Parameters without asterisks were obtained using only ADMET Predictor^®^ software. Log BB: brain/blood partition coefficient; MW: molecular weight; LogP: octanol/water partition coefficient; HAB: hydrogen acceptor bond; HDB: hydrogen donor bond; TPSA: topological polar surface; RB: rotatable bond.

**Table 3:** Prediction of the absorption, distribution, and metabolism of curcumin and its derivatives analyzed using ADMET Predictor^®^ software.

ADMET Property		Curcumin and Its Derivatives						
		1 *	2	3	4	6	11	19
**Absorption** **(A)**	Fa%	99.95%	59.74%	57.04%	73.50%	68.99%	53.70%	0.61%
	Fb%	92.39%	47.92%	51.08%	64.92%	58.54%	42.67%	0.31%
**Distribution** **(D)**	FU%	4.55%	3.00%	4.43%	4.95%	4.79%	4.29%	2.55%
	Effect. human jejunal perm. (cm/s × 10^4^)	6.55	5.70	5.16	4.71	3.23	2.86	2.45
	Skin perm. (cm/s × 10^7^)	8.82	25.80	16.35	11.72	20.17	31.82	72.33
	BBB filter	Low (97%)	Low (97%)	Low (90%)	Low (97%)	Low (97%)	Low (79%)	Low (90%)
	hERG filter	No	No	No	No	No	No	No
	P-glycoprotein inhib.	Yes (58%)	Yes (83%)	Yes (68%)	Yes (76%)	Yes (62%)	Yes (64%)	Yes (88%)
	P-glycoprotein subst.	Yes (99%)	Yes (99%)	Yes (99%)	Yes (99%)	Yes (99%)	Yes (99%)	Yes (99%)
	OATP1B1 inhib.	No (50%)	Yes (84%)	Yes (98%)	Yes (98%)	Yes (98%)	Yes (98%)	Yes (98%)
	BCRP inhib.	Yes (98%)	Yes (98%)	Yes (98%)	Yes (98%)	Yes (89%)	Yes (84%)	Yes (89%)
	BCRP subst.	Yes (95%)	Yes (64%)	Yes (95%)	Yes (84%)	Yes (95%)	Yes (95%)	Yes (53%)
**Metabolism (M)**	CYP3A4 subst.	No (54%)	No (77%)	No (33%)	Yes (70%)	No (65%)	No (66%)	Yes (73%)
	CYP3A4 inhib.	No (66%)	Yes (51%)	No (72%)	No (74%)	Yes (89%)	Yes (44%)	Yes
	CYP2D6 subst.	No (72%)	No (60%)	No (77%)	No (77%)	No (85%)	No (77%)	No (81%)
	CYP2D6 inhib.	No (84%)	No (63%)	No (84%)	No (80%)	No (84%)	No (56%)	Yes (42%)
	CYP1A2 inhib.	Yes (95%)	Yes (95%)	Yes (95%)	Yes (95%)	Yes (95%)	Yes (95%)	Yes (95%)
	CYP2C9 inhib.	No (69%)	No (61%)	Yes (34%)	No (62%)	No (67%)	No	No
	CYP2C19 inhib.	No (99%)	No (99%)	No (99%)	No (99%)	No (99%)	No (99%)	No (99%)

(*) Curcumin (**1**) was used as a control in this study. Fa%: absorbed fraction; Fb%: bioavailable fraction; FU%: fraction unbound (human); Effect. human jejunal perm.: effective human jejunal permeability; Skin perm.: skin permeability; BBB filter: blood–brain barrier filter; hERG filter: human Ether-a-go-go-Related Gene filter; P-glycoprotein inhib.: P-glycoprotein inhibitor; P-glycoprotein subst.: P-glycoprotein substrate; OATP1B1 inhib.: organic anion transporting polypeptide type 1B1 inhibitor; BCRP inhib.: breast cancer resistance protein inhibitor; BCRP subst.: breast cancer resistance protein substrate; CYP3A4 subst.: cytochrome P450 type 3A4 substrate; CYP3A4 inhib.: cytochrome P450 type 3A4 inhibitor; CYP2D6 subst.: cytochrome P450 type 2D6 substrate; CYP2D6 inhib.: cytochrome P450 type 2D6 inhibitor; CYP1A2 inhib.: cytochrome P450 type 1A2 inhibitor; CYP2C9 inhib.: cytochrome P450 type 2C9 inhibitor; CYP2C19 inhib.: cytochrome P450 type 2C19 inhibitor.

**Table 4:** Prediction of the excretion and toxicity of curcumin and its derivatives using ADMET Predictor^®^ software.

ADMET Property		Curcumin and Its Derivatives						
		1 *	2	3	4	6	11	19
**Excretion** **(E)**	OCT2 inhib.	Yes	Yes (50%)	Yes	Yes	Yes	Yes	Yes
	OCT2 subst.	No (93%)	No (84%)	No (93%)	No (93%)	No (91%)	No (82%)	No
**Toxicity** **(T)**	ORAT LD50	1439.98 mg/kg	1298.04 mg/kg	1227. 59 mg/kg	1727.98 mg/kg	1801.93 mg/kg	1143.83 mg/kg	360.75 mg/kg
	ORCT	494.67 mg/kg/day	237.63 mg/kg/day	360.51 mg/kg/day	187.56 mg/kg/day	205.83 mg/kg/day	215.78 mg/kg/day	45.13 mg/kg/day
	Max RTD	Above_3.16 (59%)	Above_3.16 (62%)	Above_3.16 (59%)	Above_3.16 (89%)	Above_3.16 (65%)	Below_3.16 (61%)	Below_3.16 (59%)
	BDG	No (95%)	No (95%)	No (95%)	No (95%)	No (83%)	No (83%)	No (95%)
	Ames Toxicity	Positive MUT m97 + 1537 (21%)	Negative	Positive MUT m97 + 1537 (21%)	Negative	Negative	Negative	Negative
	*T. pyriformis* Toxicity	0.99 mmol/L	1.69 mmol/L	0.97 mmol/L	0.91 mmol/L	0.49 mmol/L	0.48 mmol/L	1.26 mmol/L
	MT	0.07 mg/L	9 × 10^−3^ mg/L	0.04 mg/L	0.01 mg/L	0.01 mg/L	4 × 10^−3^ mg/L	1.98 × 10^−4^ mg/L
	*D. magna* Toxicity	11.47 mg/L	0.23 mg/L	4.04 mg/L	1.20 mg/L	1.23 mg/L	0.24 mg/L	0.04 mg/L
	PhL test	Nontoxic (99%)	Nontoxic (99%)	Nontoxic (99%)	Nontoxic (99%)	Nontoxic (99%)	Nontoxic (74%)	Nontoxic (84%)

(*) Curcumin (**1**) was used as a control in this study. OCT2 inhib.: organic cation transporter 2 inhibitor; OCT2 subst.: organic cation transporter 2 substrate; ORAT LD50: oral rat acute toxicity LD50; ORCT: oral rat chronic toxicity; Max RTD: maximum recommended therapeutic dose; BDG: biodegradation; Ames Toxicity: DNA mutagenic toxicity assay; *T. pyriformis* Toxicity: toxicity test with *Tetrahymena pyriformis*; MT: Minnow Toxicity; *D. magna* Toxicity: toxicity test with *Daphnia magna*; PhL test: phospholipidosis test.

**Table 5:** Prediction of the main pharmacokinetic parameters of curcumin and its derivatives (10 mg human oral dose) using ADMET Predictor^®^ software.

Pharmacokinetic Parameter	Curcumin and Its Derivatives						
	1 *	2	3	4	6	11	19
**Fa%**	99.95	59.74	57.04	73.50	68.99	53.70	0.61
**Fb%**	92.39	47.92	51.08	64.92	58.54	42.67	0.31
**Cmax (ng/mL)**	60.45	5.33	12.01	18.98	14.47	6.67	0.01
**Tmax (h)**	3.59	26.55	26.19	19.85	20.41	24.71	8.43
**AUC (ng-h/mL)**	1660.82	361.24	708.38	845.07	598.84	328.07	1.00
**Vd (L)**	137.42	645.27	274.78	224.91	240.96	346.57	1037.85
**t_1/2_ (h)**	17.15	35.37	26.89	20.39	17.12	18.53	23.93
**Cl (L/h)**	5.56	12.60	7.08	7.65	9.76	12.96	30.06

(*) Curcumin (**1**) was used as a control in this study. Fa%: absorbed fraction; Fb%: bioavailable fraction; Cmax: maximum blood concentration; Tmax: time to reach maximum blood concentration; AUC: area under the curve; Vd: volume of distribution in humans; t_1/2_: half-life; Cl: clearance.

**Table 6:** Summary of the screening results for selecting the top-performing curcumin derivatives according to their selectivity indices and in silico ADMET properties.

Compound	SI	Lipinski’s Rule	Veber	Fa%	Fb%	t_1/2_	Water Sol.	Log BB	Ames Toxicity
**11**	20.17	No	No	53.70	42.67	18.53	3 × 10^−3^	−1.24	Neg
**4**	17.16	Yes	No	73.50	64.92	20.39	5 × 10^−3^	−1.13	Neg
**6**	15.45	Yes	No	68.99	58.54	17.12	5 × 10^−3^	−1.11	Neg
**19**	13.71	No	No	0.61	0.31	18.53	1.13 × 10^−5^	−1.27	Neg
**3**	13.24	Yes	Yes	57.04	51.08	26.89	3 × 10^−3^	−0.90	Pos
**2**	3.0	Yes	Yes	59.74	47.92	35.37	4 × 10^−3^	−0.84	Neg
**1**	2.52	Yes	Yes	99.95	92.39	17.00	0.05	−0.82	Pos

The compounds are ranked as follows in order of predicted ADMET performance (best to worst): **1**, **3**, **2**, **4**, **6**, **11**, and **19**.

## Data Availability

Data are contained within the article and Supplementary Material.

## Supplementary Materials

The following supporting information can be downloaded at: https://www.mdpi.com/article/10.3390/pharmaceutics17080968/s1, Figure S1: Methodology of synthesis of Curcumin derivatives (**2**); Figure S2: Methodology of synthesis of Curcumin derivatives (**3**–**4**); Figure S3: Methodology of synthesis of Curcumin derivatives (**5**–**22**).
