# Supplementary material for: Insights into the Structural Patterns in Human Glioblastoma Cell Line SF268 Activity and ADMET Prediction of Curcumin Derivatives
Source: Pharmaceutics. 2025 Jul 25;17(8):968. doi: 10.3390/pharmaceutics17080968 (PMC12389136; doi:10.3390/pharmaceutics17080968)

SUPPORT INFORMATION

Article

Article

Insights into the Structural Patterns in Human Glioblastoma Cell Line SF268 Activity and ADMET Prediction of Curcumin Derivatives

Lorena Coronado [1,2,*], Johant Lakey-Beitia [2,3,*], Marisin Pecchio [4], Michelle G. Ng [1], Ricardo Correa [1,2], Gerardo Samudio-Ríos [1,5], Jessica Cruz-Mora [3], Arelys L. Fuentes [3], K. S. Jagannatha Rao [6] and Carmenza Spadafora [1,2]

1 Center for Molecular and Cellular Biology of Diseases, Instituto de Investigaciones Científicas y Servicios de Alta Tecnología (INDICASAT AIP), Clayton, City of Knowledge, Panama City 0843-01103, Panama; mng@indicasat.org.pa (M.G.N.); rcorrea@indicasat.org.pa (R.C.); gerardo.samudio@utp.ac.pa (G.S.-R.); cspadafora@indicasat.org.pa (C.S.)
2 Sistema Nacional de Investigación (SNI), SENACYT, Panama City 0816-02852, Panama
3 Center for Biodiversity and Drug Discovery, Instituto de Investigaciones Científicas y Servicios de Alta Tecnología (INDICASAT AIP), Clayton, City of Knowledge, Panama City 0843-01103, Panama; jcruz@indicasat.org.pa (J.C.-M.); afuentes@indicasat.org.pa (A.L.F.)
4 Center for Academic Affairs and Collaboration, Instituto de Investigaciones Científicas y Servicios de Alta Tecnología (INDICASAT AIP), Clayton, City of Knowledge, Panama City 0843-01103, Panama; mpecchio@indicasat.org.pa
5 PhD Program in Biosciences and Biotechnology, Faculty of Science and Technology, Universidad Tecnológica de Panamá, Panama City 0819-07289, Panama
6 Department of Biotechnology, Koneru Lakshmaiah Education Foundation (KLEF) Deemed to be University, Vaddeswaram 522302, India; prochancellor@kluniversity.in
* Correspondence: lcoronado@indicasat.org.pa (L.C.); jlakey@indicasat.org.pa (J.L.-B.); Tel.: +507-517-0700 (L.C. & J.L.-B.)

Synthetic Scheme of compounds reactions

Synthetic Scheme of Monoalkylether-curcumin for compound **2**

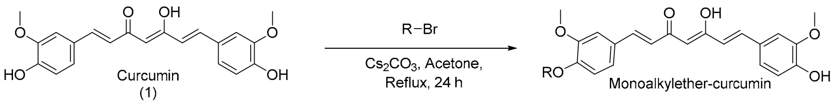

Supplementary Figure S1. Methodology of synthesis of Curcumin derivatives (**2**)

Synthetic Scheme of Monoalkylcurcumin and dialkylcurcumin Derivatives for compounds **3-4**

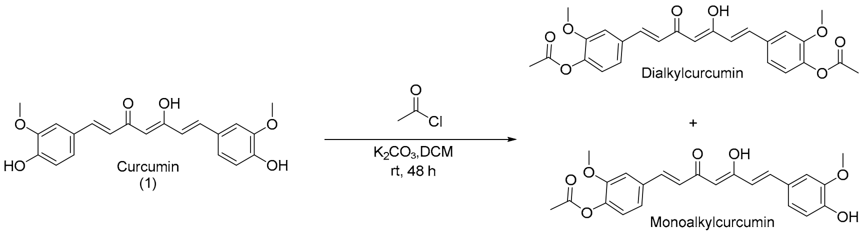

Supplementary Figure S2. Methodology of synthesis of Curcumin derivatives (**3-4**)

Synthetic Scheme of Monoalkylsuccinyl-curcumin and dialkylsuccinyl-curcumin Derivatives for compound **5-22**.

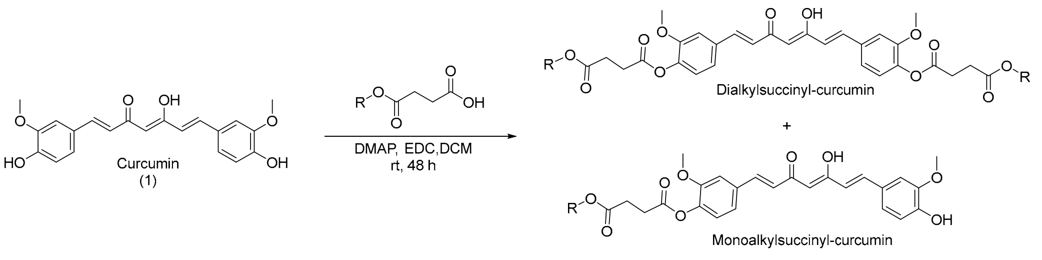

Supplementary Figure S3. Methodology of synthesis of Curcumin derivatives (**5-22**)

(1*E*,3*Z*,6*E*)-3-hydroxy-5-oxohepta-1,3,6-triene-1,7-diyl)bis(2-methoxy-4,1-phenylene)diacetate (4)

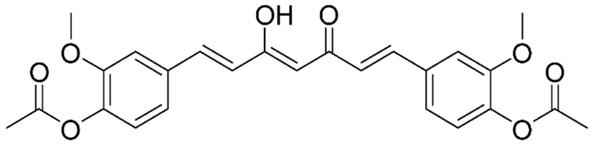

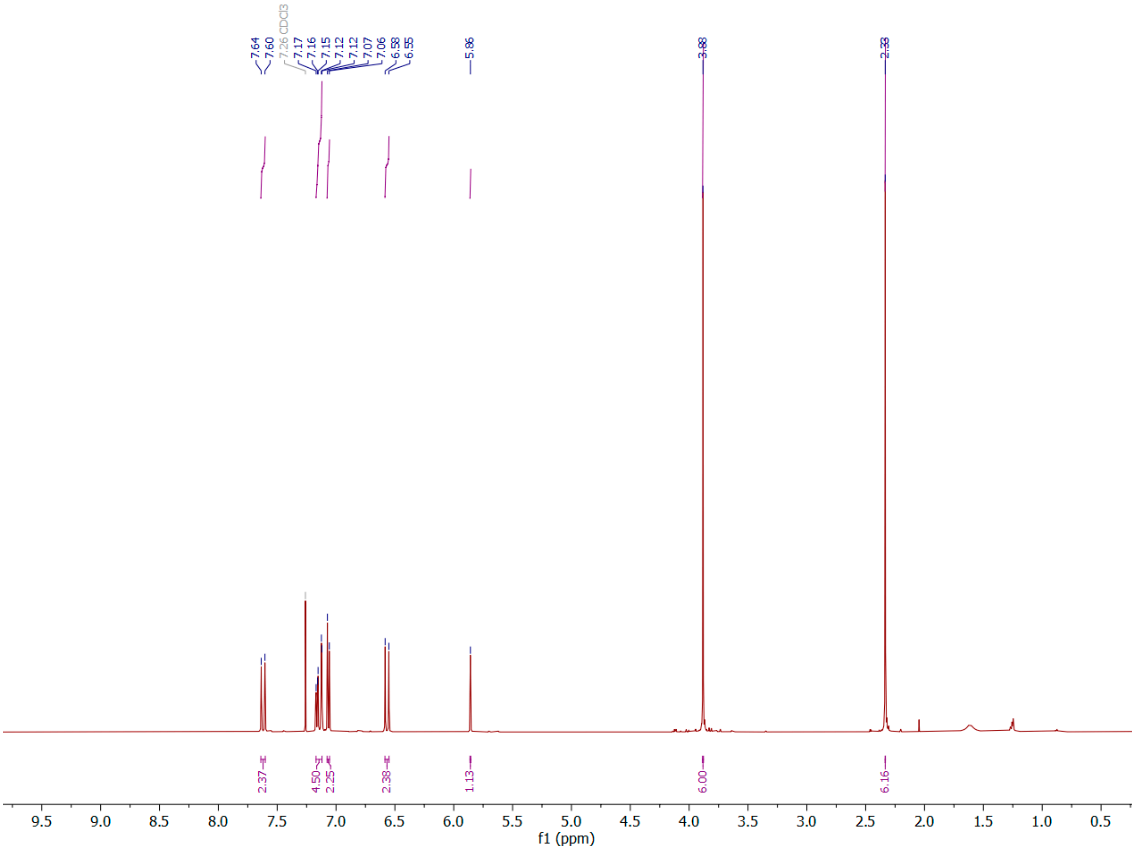

(1*E*,3*Z*,6*E*)-3-hydroxy-5-oxohepta-1,3,6-triene-1,7-diyl)bis(2-methoxy-4,1-phenylene)diacetate (4)

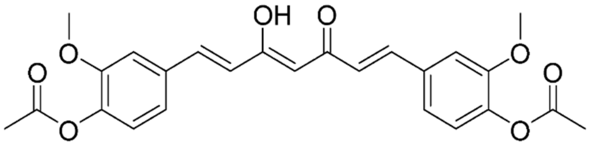

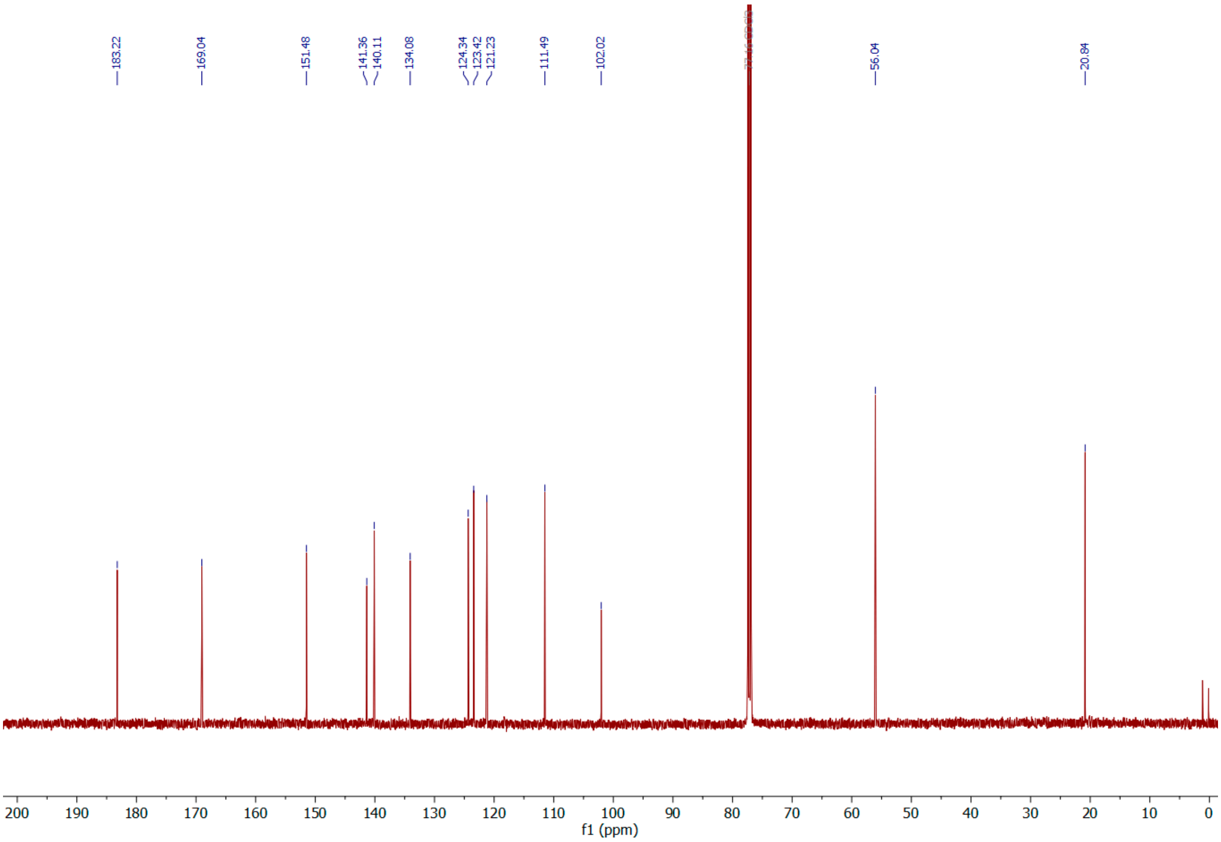

Cyclopentyl (4-((1*E*,3*Z*,6*E*)-3-hydroxy-7-(4-hydroxy-3-methoxyphenyl)-5-oxohepta-1,3,6-trien-1-yl)-2-methoxyphenyl) succinate (5).

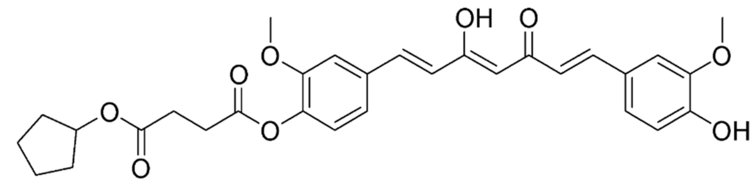

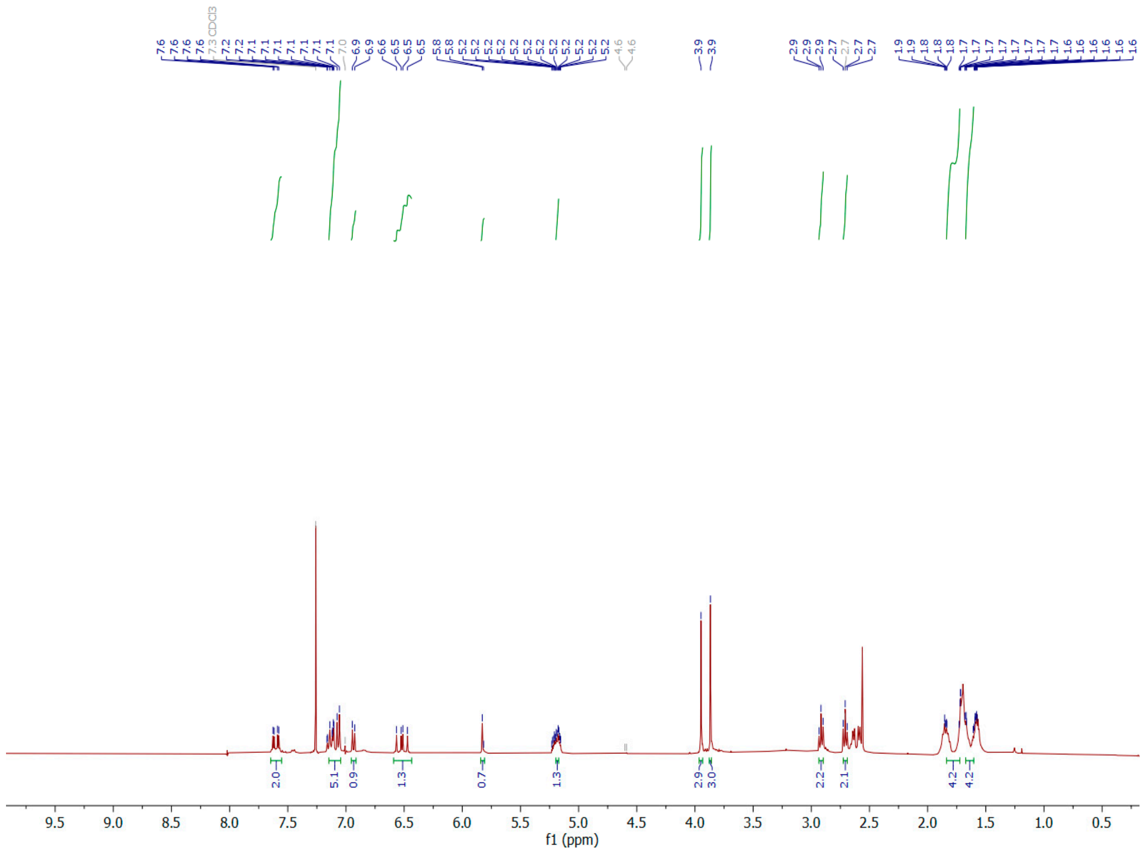

Cyclopentyl (4-((1*E*,3*Z*,6*E*)-3-hydroxy-7-(4-hydroxy-3-methoxyphenyl)-5-oxohepta-1,3,6-trien-1-yl)-2-methoxyphenyl) succinate (5)

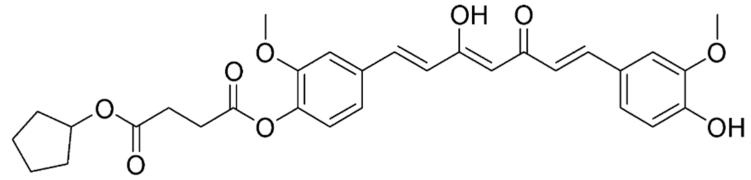

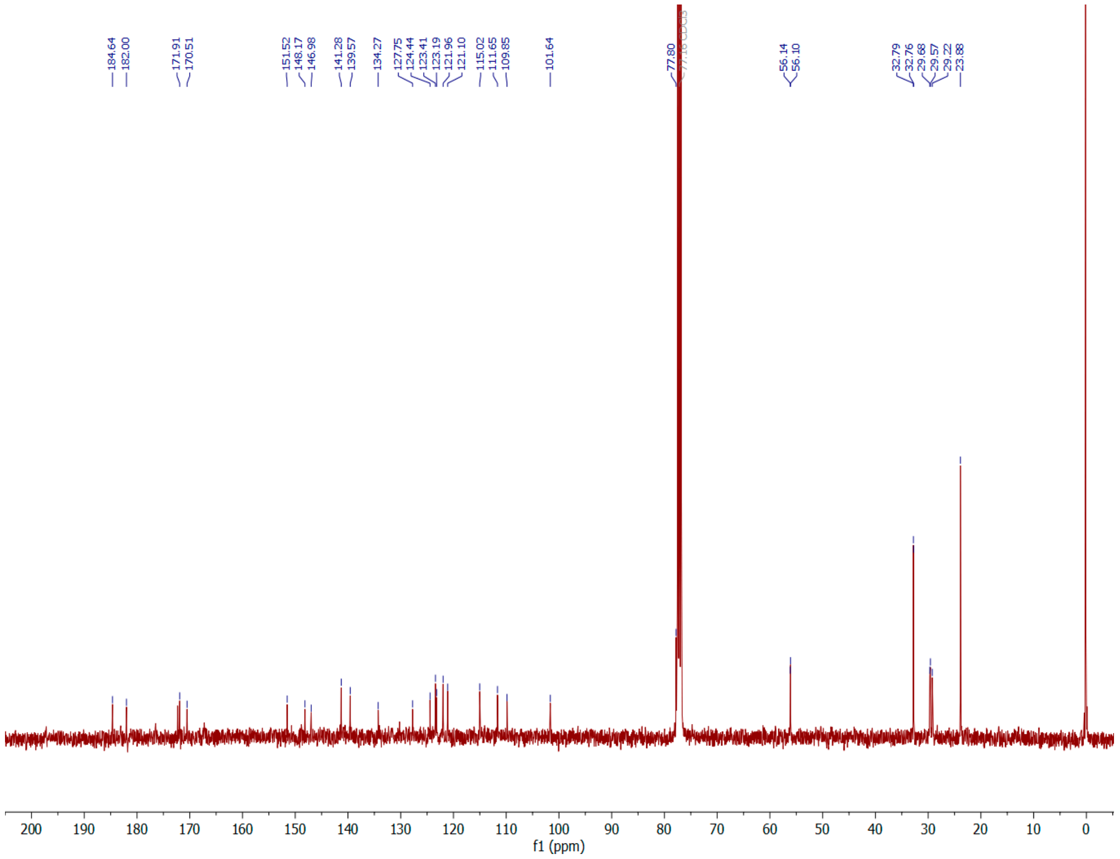

Allyl (4-((1*E*,3*Z*,6*E*)-3-hydroxy-7-(4-hydroxy-3-methoxyphenyl)-5-oxohepta-1,3,6-trien-1-yl)-2-methoxyphenyl) succinate (11)

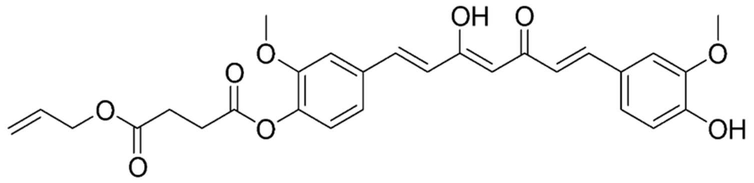

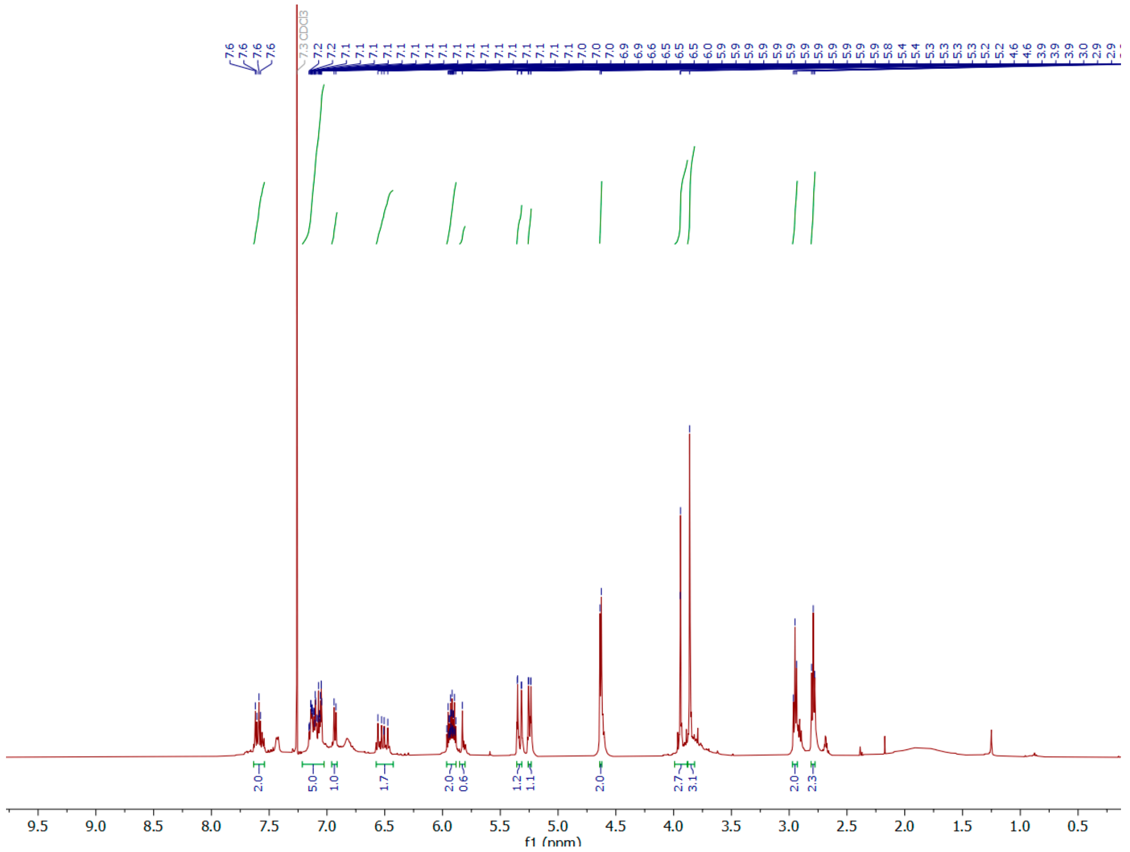

Allyl (4-((1*E*,3*Z*,6*E*)-3-hydroxy-7-(4-hydroxy-3-methoxyphenyl)-5-oxohepta-1,3,6-trien-1-yl)-2-methoxyphenyl) succinate (11)

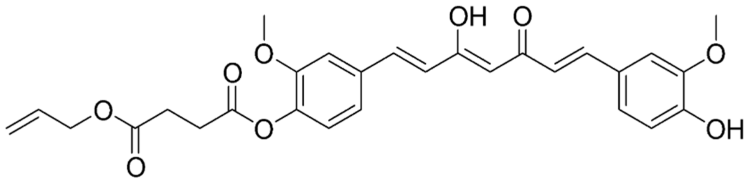

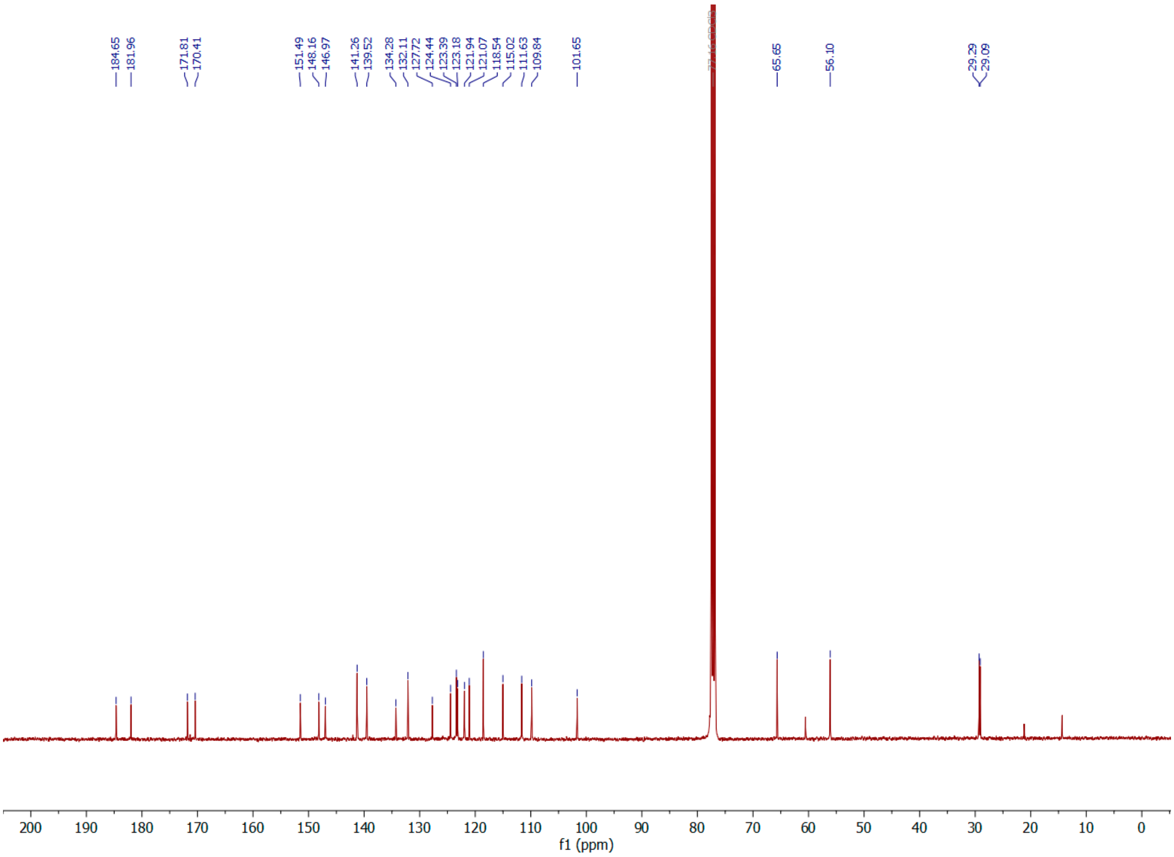

Diallyl *O*,*O*'-(((1*E*,3*Z*,6*E*)-3-hydroxy-5-oxohepta-1,3,6-triene-1,7-diyl)bis(2-methoxy-4,1-phenylene)) disuccinate (12)

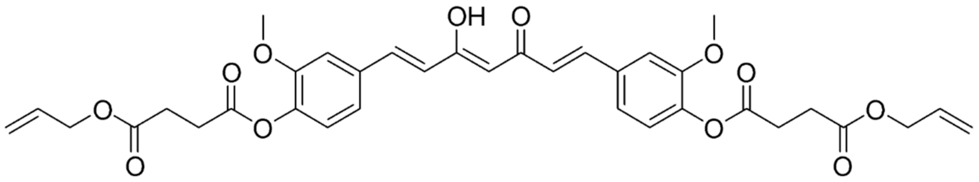

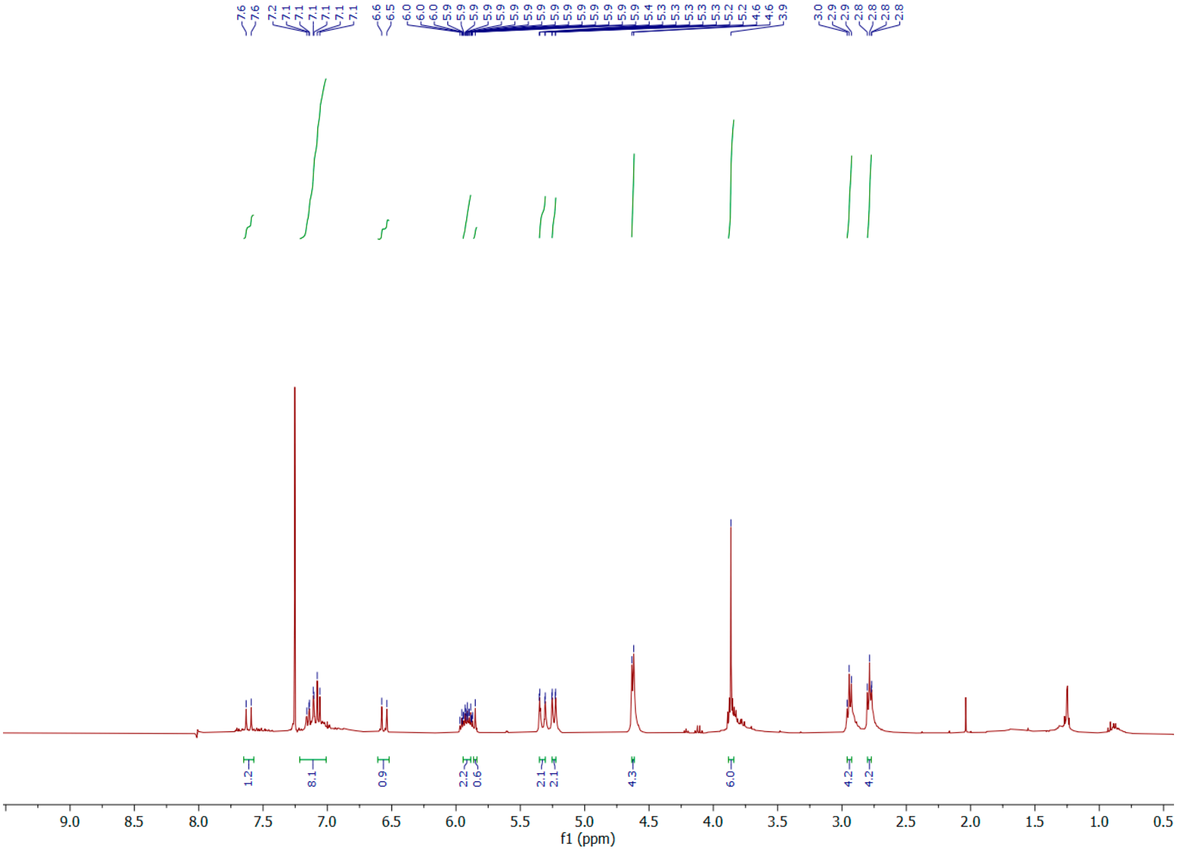

Diallyl *O*,*O*'-(((1*E*,3*Z*,6*E*)-3-hydroxy-5-oxohepta-1,3,6-triene-1,7-diyl)bis(2-methoxy-4,1-phenylene)) disuccinate (12)

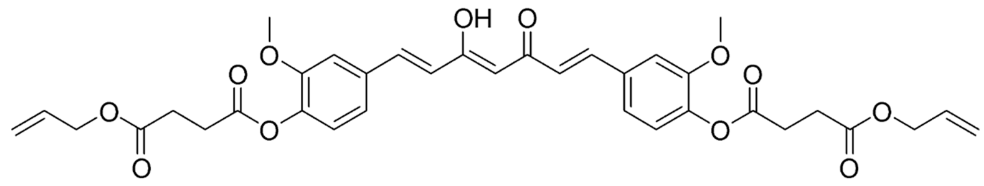

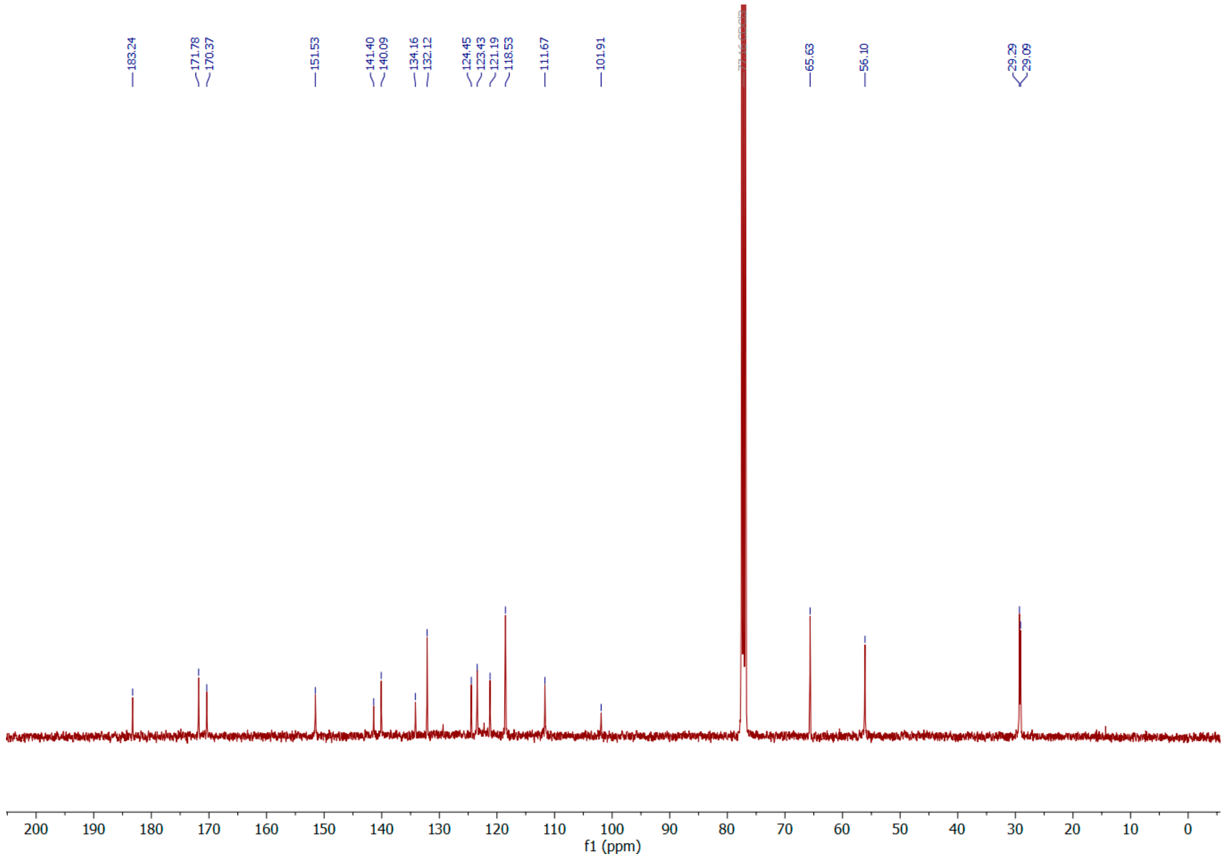

Benzyl (4-((1*E*,3*Z*,6*E*)-3-hydroxy-7-(4-hydroxy-3-methoxyphenyl)-5-oxohepta-1,3,6-trien-1-yl)-2-methoxyphenyl) succinate (13).

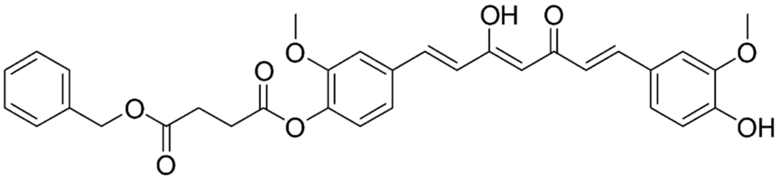

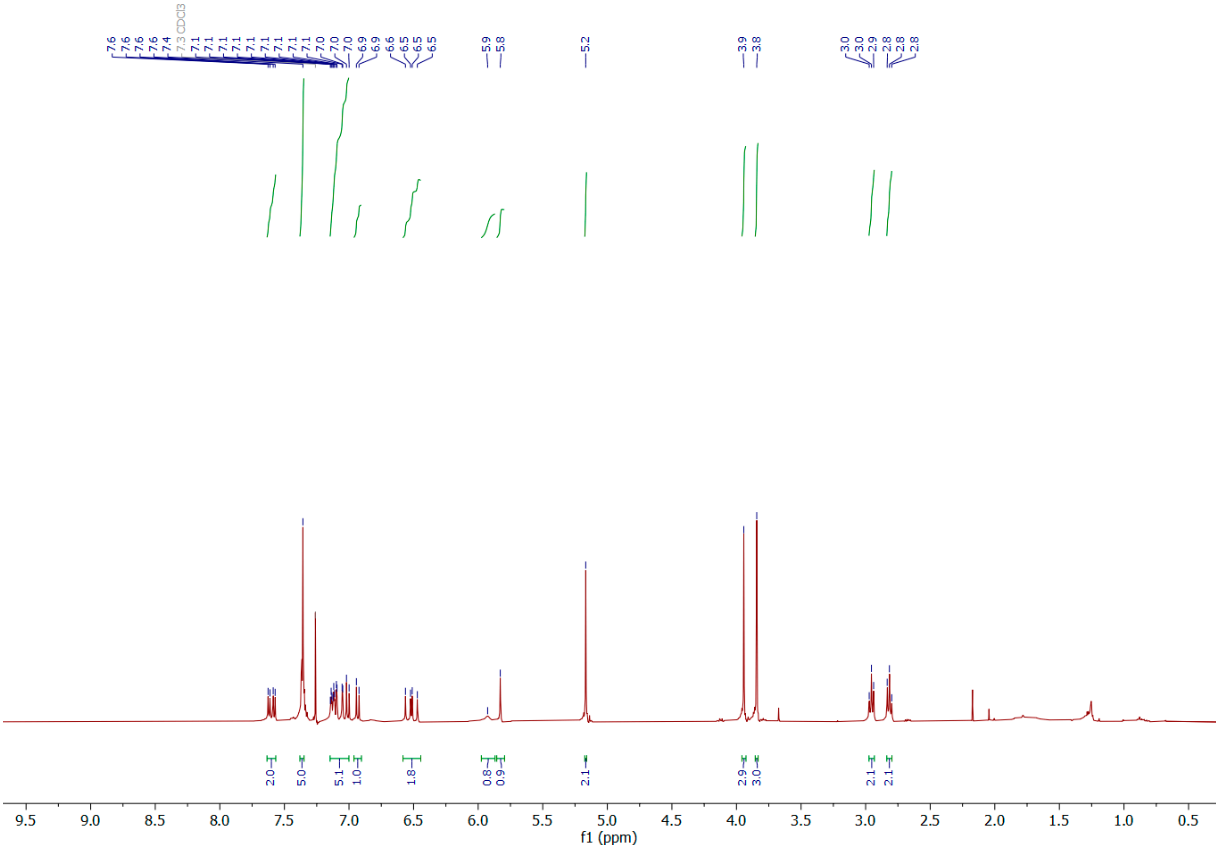

Benzyl (4-((1*E*,3*Z*,6*E*)-3-hydroxy-7-(4-hydroxy-3-methoxyphenyl)-5-oxohepta-1,3,6-trien-1-yl)-2-methoxyphenyl) succinate (13).

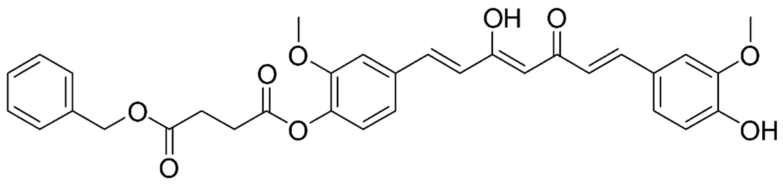

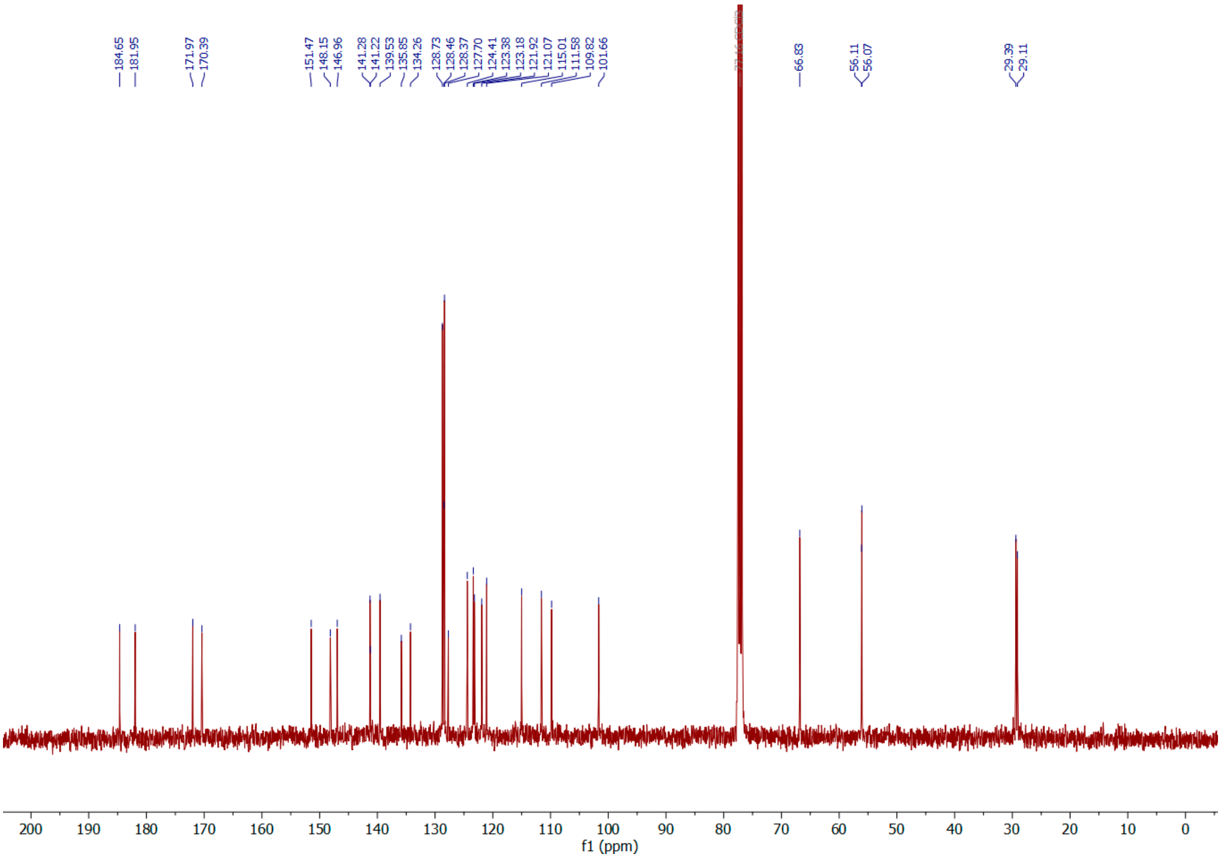

Dibenzyl *O*,*O*'-(((1*E*,3*Z*,6*E*)-3-hydroxy-5-oxohepta-1,3,6-triene-1,7-diyl)bis(2-methoxy-4,1-phenylene)) disuccinate (14)

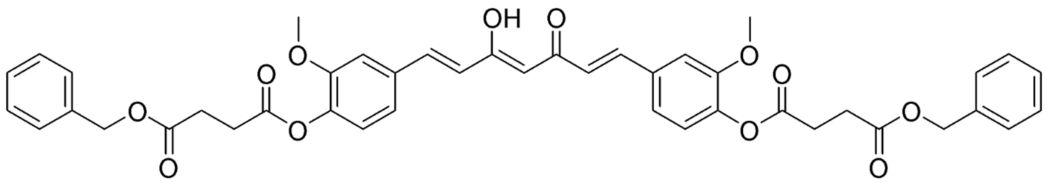

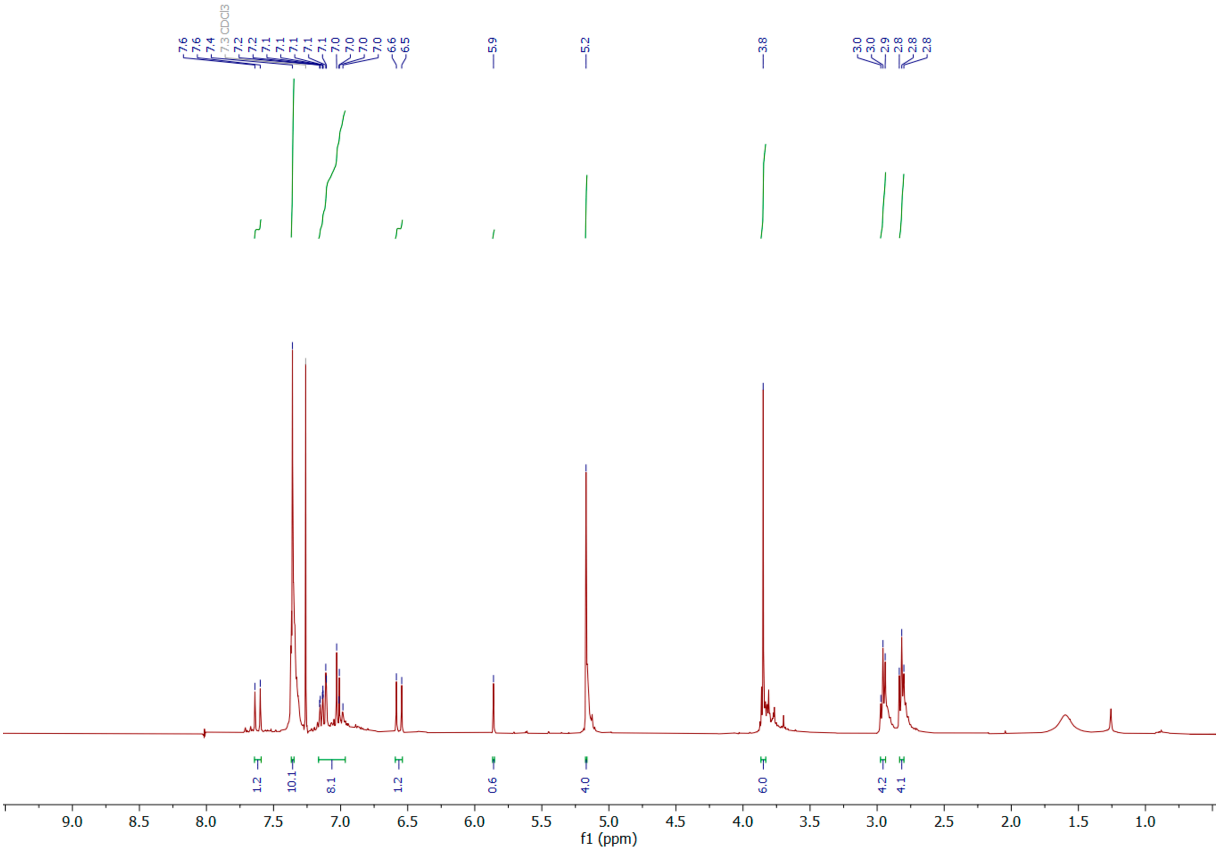

Dibenzyl *O*,*O*'-(((1*E*,3*Z*,6*E*)-3-hydroxy-5-oxohepta-1,3,6-triene-1,7-diyl)bis(2-methoxy-4,1-phenylene)) disuccinate (14)

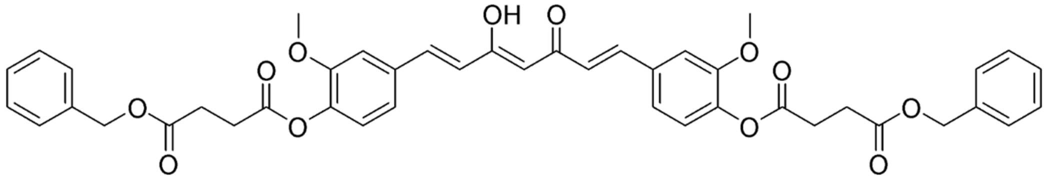

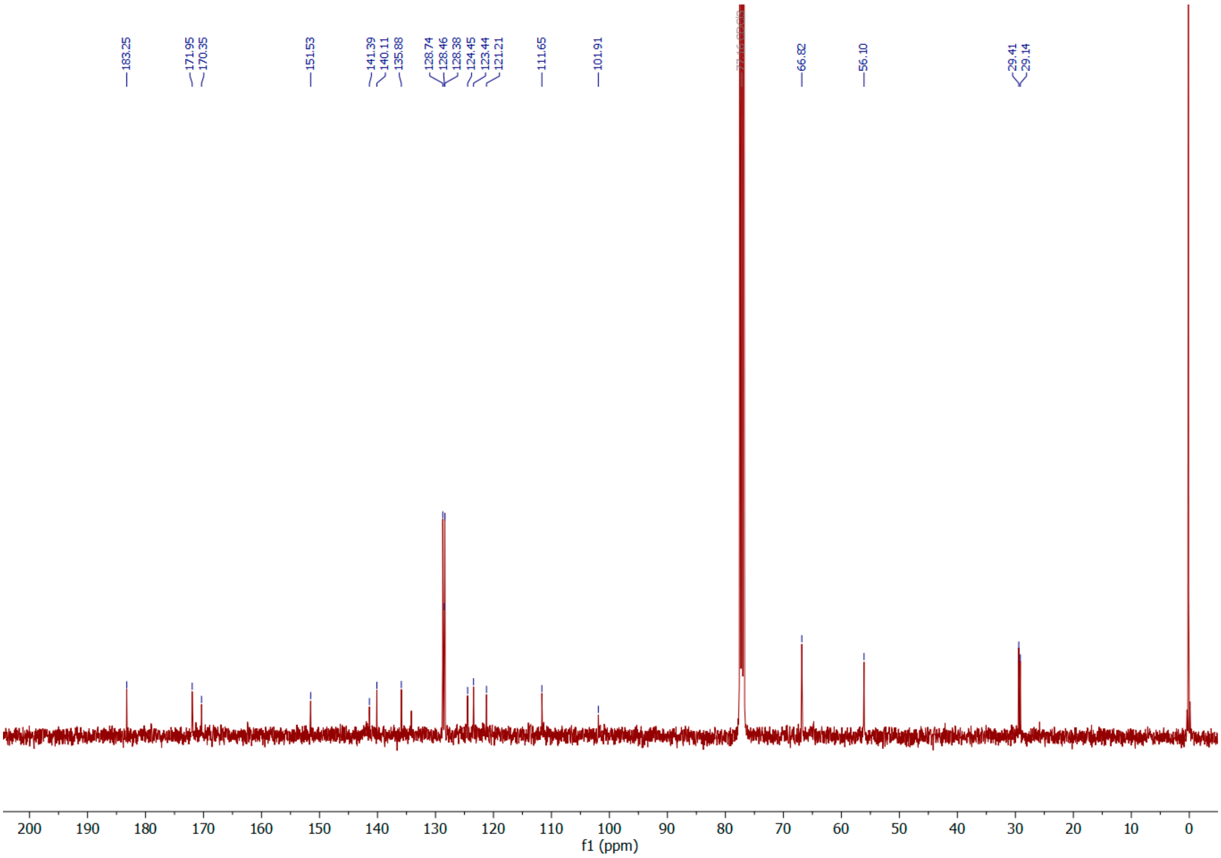

Supplement: Supplementary file 1 [file pharmaceutics-17-00968-s001.zip › pharmaceutics-3733580-supplementary.pdf]
